# Steroidal Saponins from Water Eggplant (Fruits of *Solanum torvum*) Exhibit Anti-Epileptic Activity against Pentylenetetrazole-Induced Seizure Model in Zebrafish

**DOI:** 10.3390/molecules29061316

**Published:** 2024-03-15

**Authors:** Rui Ren, Ming-yan Zhang, Tengyun Shu, Ya-ting Kong, Li-hua Su, Hai-zhou Li

**Affiliations:** Faculty of Life Science and Technology, Kunming University of Science and Technology, 727 Jingming South Road, Chenggong District, Kunming 650500, China; 20212118088@stu.kust.edu.cn (R.R.); 20192118072@stu.kust.edu.cn (M.-y.Z.); tengyunshu@163.com (T.S.); kustkongyt@163.com (Y.-t.K.)

**Keywords:** *Solanum torvum*, water eggplant, steroidal saponin, anti-epileptic activity, hepatotoxicity

## Abstract

The fruits of *Solanum torvum* Swartz, a wild relative of eggplant, are consumed as a wild vegetable in tropical regions of Africa, Asia, and South America. In traditional Chinese medicine, it is believed to have anti-inflammatory and sedative effects. In the Philippines, water decoction is used to treat hyperactivity disorder. Twenty-two steroidal saponins were isolated and purified from the fruits grown in Yunnan, China, including six new compounds: torvosides U–Z (**1**–**6**). During drying and cooking, the saponins may undergo transformation, resulting in small amounts of sapogenins. These transformations can include dehydration of hydroxyl groups at position C22, formation of double bonds at position 20, 22 or 22, 23, and even formation of peroxide products. Saponin compounds torvoside X (**4**), torvoside Y (**5**), torvoside A (**7**), and (25*S*)-3-oxo-5*α*-spirostan-6*α*-yl-*O*-*β*-d-xylopyranoside (**20**), which are glycosylated at C-6, showed certain anti-epileptic activity in a pentylenetetrazole-induced zebrafish seizure model. No antiproliferative activity was detected when tested on the cancer cell line HepG2, and no hepatotoxic effect was noted on normal liver cell line LO2.

## 1. Introduction

*Solanum torvum* Swartz is a subshrub from the Solanaceae family, which is widespread in tropical regions of Africa, Asia, and South America. The fruit of *S. torvum*, commonly known as water eggplant (also turkey berry, bhankatiya, sundakkai, kudanekayi), is an edible vegetable variety, which is rich in various beneficial components. In Yunnan Province, located in southwestern China, as well as in southeast Asian countries, such as Thailand, Malaysia, and the Philippines, many ethnic minority areas have a long-standing tradition of consuming young fruits of *S. torvum*. For instance, the Va people in Yunnan use bamboo pestles to mash the fruit into a paste, which is then seasoned with salt and chili to make a side dish. Similarly, the Jingpo people in Ruili, Yunnan, cook the fruit with pickled bamboo shoots and seasoning, or crush the cooked fruit and mix it with pickled bamboo shoots. In Thailand, fried crispy bhankatiya (the Thai name for the fruit of *S. torvum*) curry is a popular dish among international tourists.

However, water eggplant is not only used as food. It has long been employed in traditional medicine in Africa and Asia for the prevention and treatment of various ailments [1]. The investigation into the chemical constitution of *S. torvum* can be traced back to the 1940s, with studies revealing the presence of steroidal saponins, flavonoids, organic acids, and other structural classifications [2,3,4,5,6,7]. Pharmacological investigations have unveiled the antioxidant [8], antidiabetic [9], antidepressant [10], antihypertensive [11], and antitumor [12] properties of select sterols and sterol glycosides derived from *S. torvum*. Due to its calming, digestive, hemostatic, and diuretic attributes, *S. torvum* is frequently employed in traditional medicinal practices in southern China and southeast Asia.

Epilepsy is a chronic neurological disorder, which affects approximately 65 million people worldwide. It is caused by abnormal discharges of groups of neurons, mainly due to an imbalance between excitatory and inhibitory neural transmission [13]. Traditional Chinese medicine has been found to have significant efficacy in treating epilepsy, with lower toxicity and fewer side effects, providing a significant advantage over conventional treatments [14]. Recent investigations have indicated that the methanol extract of water eggplant, employed as an anti-epileptic and antispasmodic medication in traditional Philippine medicine, contains steroidal saponins, which exhibit anticonvulsant effects [7]. Consequently, the research on water eggplant has garnered heightened significance due to the utilization of its medicinal and edible resources. This study aims to isolate and purify the medicinal components of Yunnan water eggplant. The goal is to identify new structures of steroidal saponins, which can effectively treat epilepsy. This could potentially allow for the dual use of water eggplant as both a food and a medicinal product.

## 2. Results

### 2.1. Structural Elucidation

**Figure 1:**
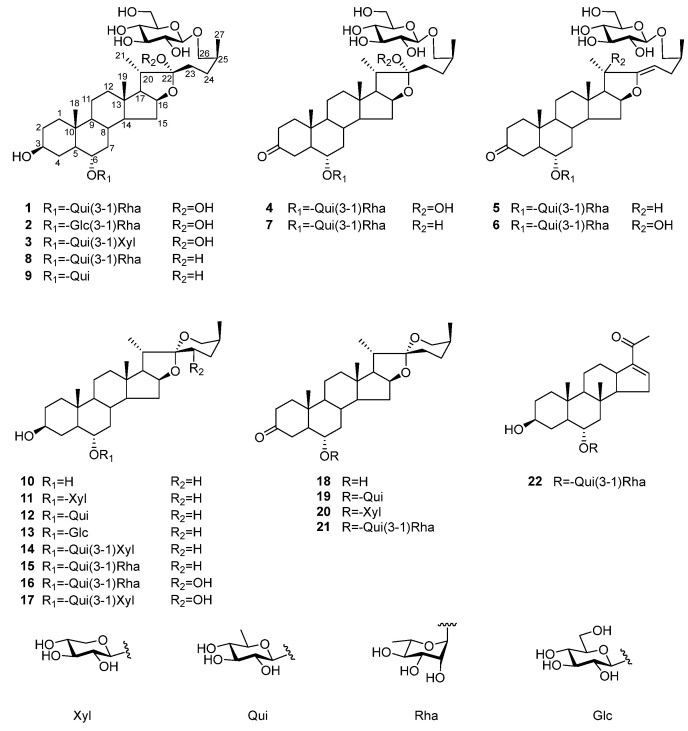
Chemical structure of constituents.

The extraction of *Solanum torvum* fruit was separated using silica gel, octadecylsilyl silica gel (ODS), and preparative HPLC to afford six new steroidal saponins, namely torvosides U–Z (**1**–**6**), together with sixteen known compounds (shown in Figure 1).

**Figure 2:**
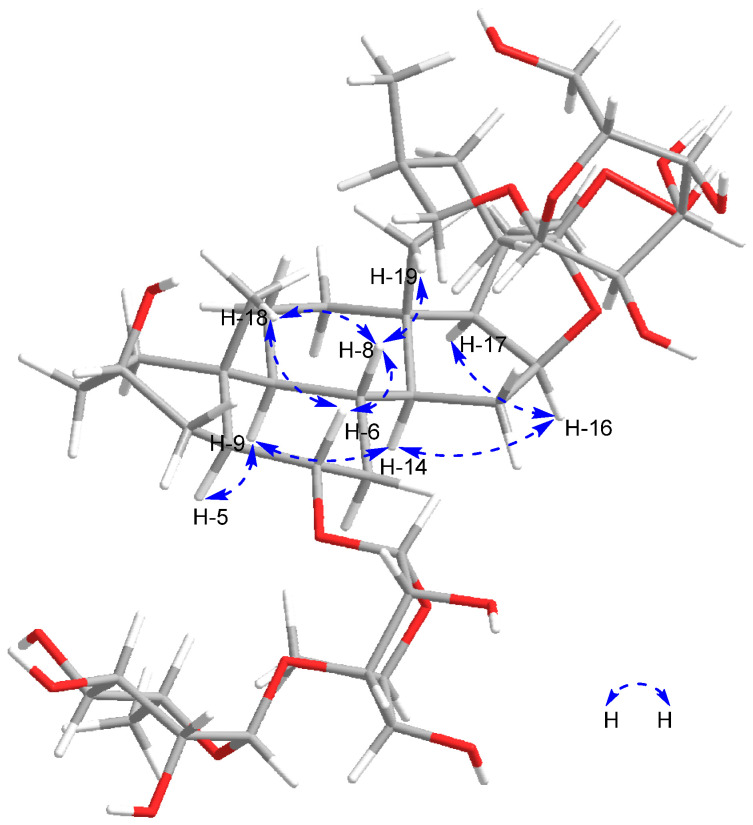
The key ROESY correlations of compound **1**.

Compound **1** (358 mg), a white amorphous powder, is readily dissolved in methanol. The anisaldehyde reagent reaction exhibits a yellow-green color, while the Ehrlich reagent reaction shows a red color, suggesting that the compound may be a furan steroid compound. (+)-HR-ESI-MS reveals an ion peak at *m*/*z* 943.4892 [M + Na]^+^, and in combination with NMR spectroscopic data, its molecular formula is determined to be C_45_H_76_O_19_ (the calculated value for C_45_H_76_O_19_Na is 943.4878). The ^1^H NMR (Table 1) spectrum of **1** displays signals for two angular methyl groups at *δ*_H_ 0.81 (3H, s, H_3_-18) and 0.80 (3H, s, H_3_-19), two methyl doublet signals at *δ*_H_ 1.10 (3H, d, *J* = 5.6 Hz, H_3_-27) and *δ*_H_ 1.25 (3H, d, *J* = 6.0 Hz, H_3_-21), one oxygen bearing methine proton signal at *δ*_H_ 3.63 (1H, m, H-6), and two oxymethylene proton signals at *δ*_H_ 3.53 and 4.16 (each 1H, m, H-26a, H-26b), which correlate in the HSQC spectrum (shown in Supplementary Materials) with *δ*_H_ 75.6, indicating a glycosyl substitution at C-26 characteristic of furostanol-type saponins. The A/B *trans*-ring fusion is confirmed by observation of signals in the ROESY spectra (Figure 2) at *δ*_H_ 1.26 (H-5), 0.54 (H-9), 0.95 (H-14), 4.77 (H-16), and 1.80 (H-17), indicating that the aglycone is a 5*α*-steroidal sapogenin [15]. According to the absolute configuration of C-25 in the literature [12], if the difference in chemical shift between the two hydrogen atoms on the carbon at C-26 is Δab > 0.57 ppm, it is determined to be the 25*S* configuration, and when Δab < 0.48 ppm, it is determined to be the 25*R* configuration. The chemical shift in hydrogen atoms at *δ*_H_ 4.16 (H-26a) and 3.53 (H-26b) in this compound is Δab = 0.63 > 0.57 ppm, indicating that C-25 is in the *S* configuration. The ROESY (shown in Supplementary Materials) cross peaks between *δ*_H_ 0.81 (H_3_-18)/*δ*_H_ 1.60 (H-8), 1.60 (H-8)/3.66 (H-6), and 3.66 (H-6)/0.80 (H_3_-19) elucidate the stereochemistry of the aglycone, indicating the *ꞵ*-axial orientation of H-6 and, consequently, the *α*-equatorial orientation of 6-OH. The ^13^C NMR (Table 2) and HSQC spectra (shown in Supplementary Materials) portray 45 carbon signals of which 18 are assigned to the sugar moieties and 27 to the aglycone, including 4 methyl groups at *δ*_C_ 13.9 (C-18), 16.7 (C-19), 16.2 (C-21), 17.9 (C-27), and the downfield quaternary carbon signal at *δ*_C_ 82.6 (C-16). The chemical shifts are consistent with literature data on torvoside A [4] and include three sugar anomeric carbon signals at *δ*_C_ 105.9, 105.6, and 103.4, corresponding to a quinovose, a rhamnose, and a glucose. The molecular weight of compound **1** is 16 times higher than that of torvoside A, corresponding to the molecular weight of an oxygen atom, and *δ*_C_ 117.6 (C-22) shows a downward shift, indicating that compound **1** may have a peroxide hydroxyl group at C-22. According to the method [16], compound **1** was reacted with triphenylphosphine, which reduced the peroxide hydroxyl group to a hydroxyl group. The product exhibited an Rf value of 0.357, consistent with that of the torvoside A standard.

**Figure 3:**
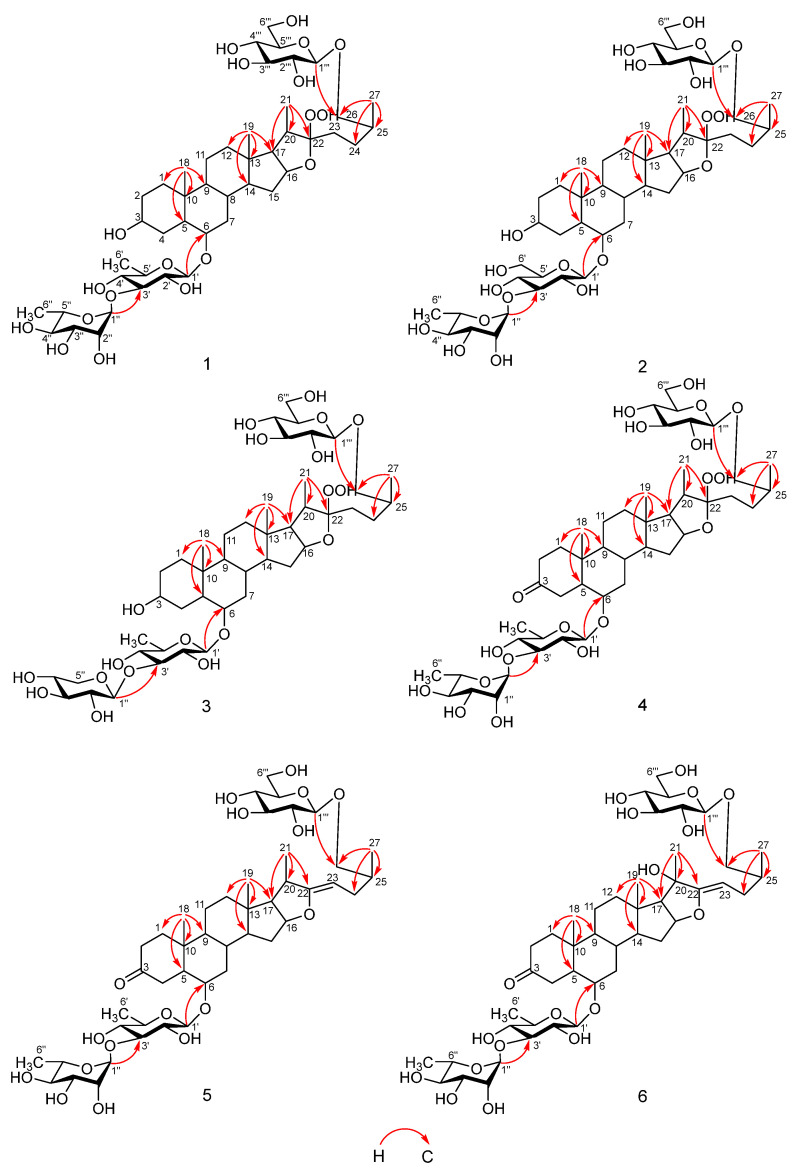
The key HMBC correlations of compounds **1**–**6.**

In the HMBC spectrum (Figure 3), the correlation between *δ*_H_ 4.80 (d, *J* = 6.0 Hz, H_1_-1′) and *δ*_C_ 79.4 (C-6), *δ*_H_ 6.36 (br s, H_1_-1″) and *δ*_C_ 83.3 (C-3′), and *δ*_H_ 4.86 (d, *J* = 7.6 Hz, H_1_-1′″) and *δ*_C_ 75.6 (C-26) indicates the glycosylation site in compound **1**, with glucose linked at C-26, quinovose connected at C-6, and rhamnose connected at the C-3 position of quinovose. In addition, the correlations between *δ*_H_ 1.10 (d, *J* = 5.6 Hz, H_3_-27) and *δ*_C_ 28.7 (C-24), 34.9 (C-25), and 75.6 (C-26), between *δ*_H_ 1.25 (d, *J* = 6.0 Hz, H_3_-21) and *δ*_C_ 64.1 (C-17), 40.4 (C-20), and 117.6 (C-22), and between *δ*_H_ 0.80 (3H, s, H_3_-19) and *δ*_C_ 40.1 (C-12), 41.6 (C-13), 56.4 (C-14), and 64.1 (C-17) further prove its structure, confirming that compound **1** is a new compound named torvoside U.

Compound **2** (50.9 mg) is a white amorphous powder, which readily dissolves in methanol. The anisaldehyde reagent and Ehrlich reagent yield positive results, suggesting the presence of a furan steroid compound. The (+)-HR-ESI-MS exhibits an ion peak at *m*/*z* 959.4833 [M + Na]^+^, indicating a likely molecular formula of C_45_H_76_O_20_ (calculated as C_45_H_76_O_20_Na: 959.4828). The ^1^H-NMR (pyridine-*d*_5_, 600 MHz) data reveal two distinct single-peak methyl signals at *δ*_H_ 0.82 (3H, s, H_3_-18) and 0.77 (3H, s, H_3_-19), along with two double-peak methyl signals at *δ*_H_ 1.10 (d, *J* = 6.5 Hz, H_3_-27) and 1.23 (d, *J* = 7.0 Hz, H_3_-21). These four signals are indicative of characteristic methyl proton signals for steroid saponins. Additionally, there is a relatively high-field double-peak methyl signal at *δ*_H_ 1.72 (d, *J* = 6.5 Hz, H_3_-6″). The ^13^C-NMR (pyridine-*d*_5_, 150 MHz) data demonstrate that compound **2** comprises 45 carbon signals, including 3 sugar anomeric carbon signals *δ*_C_ 106.2, 105.6, and 103.3. A comparison of the signals with those of compound **1**, except for the sugar unit, reveals highly consistent chemical shift values. Hence, it can be inferred that the C-22 of compound **2** also possesses a peroxide hydroxyl configuration. Additionally, the chemical signals of *δ*_C_ 79.8 (C-6) and 75.6 (C-26) suggest that C-6 and C-26 may serve as sites of glycosylation. The NMR sugar unit chemical shift values of compound **2** are in agreement with those of the known compound (25*S*)-26-(*β*-D-glucopyranosyloxy)-3*β*-hydroxy-22*α*-methoxy-5*α*-furostan-6*α*-yl-*O*-*α*-L-rhamnopyranosyl-(1→3)-*β*-D-glucopyranoside [6].

In the HMBC spectrum (Figure 3), the correlation between *δ*_H_ 4.88 (Glc II H-1) and *δ*_C_ 75.6 (C-26) in HMBC and the correlation between *δ*_H_ 3.52 (H-26a) and *δ*_C_ 105.6 (Glc II C-1) demonstrate the connection between Glc II and glycone C-26. The correlation between *δ*_H_ 4.85 (Glc I H-1) and *δ*_C_ 79.8 (C-6) indicates the connection between Glc I and C-6, while the correlation between *δ*_H_ 4.07 (Glc I H-2) and *δ*_C_ 83.5 (Glc I H-3) and the correlation between *δ*_H_ 6.39 (Rha H-1) and *δ*_C_ 83.5 (Glc I C-3′) confirm the connection between Rha and Glc I C-3′. Furthermore, the correlation between *δ*_H_ 1.10 (d, *J* = 6.5 Hz, H_3_-27) and *δ*_C_ 28.7 (C-24), 34.9 (C-25), and 75.6 (C-26), the correlation between *δ*_H_ 1.23 (d, *J* = 7.0 Hz, H_3_-21) and *δ*_C_ 64.3 (C-17), 40.4 (C-20), and 117.6 (C-22), the correlation between *δ*_H_ 0.77 (3H, s, H_3_-19) and *δ*_C_ 41.6 (C-13), 56.4 (C-14), and 64.3 (C-17), and the correlation between *δ*_H_ 0.82 (3H, s, H_3_-18) and *δ*_C_ 54.1 (C-9), 37.0 (C-10) provide further evidence of its structure. Moreover, the *δ*_H_ 4.15 (H-26a), 3.52 (H-26b) with Δab = 0.63 > 0.57 ppm confirms the 25*S* configuration. Therefore, the compound is determined to be a novel compound and is named torvoside V.

Compound **3** (11.4 mg), a white powdery substance, dissolves in methanol. Positive results were obtained with the anisaldehyde reagent and Ehrlich reagent. (+)-HR-ESI-MS exhibited *m*/*z* 929.4733 [M + Na]^+^, confirming its molecular formula as C_44_H_74_O_19_ (calculated as C_44_H_74_O_19_Na: 929.4722). The ^1^H-NMR (pyridine-*d*_5_, 600 MHz) spectra revealed two single-peak methyl signals at *δ*_H_ 0.81 (3H, s, H_3_-18) and 0.80 (3H, s, H_3_-19), as well as two double-peak methyl signals at 1.10 (d, *J* = 6.5 Hz, H_3_-27) and 1.25 (d, *J* = 6.8 Hz, H_3_-21). These distinct methyl proton signals indicate the presence of a steroid saponin. The ^13^C-NMR (pyridine-*d*_5_, 150 MHz) spectra showed 44 carbon signals for compound **3**, including 3 sugar anomeric carbon signals at *δ*_C_ 105.5, 105.6, and 106.9. A comparison of the chemical signals of compound **3** with compound **1** suggests the presence of the same aglycone moiety. Additionally, the chemical shift values of sugar unit signals closely matched those of the known compound macaoside L [17]. In the HMBC spectrum (Figure 3), correlations were observed between *δ*_H_ 4.86 (d, *J* = 7.1 Hz, H-1′) and *δ*_C_ 79.4 (C-6), *δ*_H_ 5.30 (d, *J* = 7.4, H-1″) and *δ*_C_ 87.9 (C-3′), and *δ*_H_ 4.87 (H-1″′) and *δ*_C_ 75.6 (C-26), indicating the connection positions of the sugars in compound **3**. Specifically, glucose was connected at C-26, quinovose at C-6, and xylose at C-3 of quinovose. Based on these findings, the structure of compound **3** was determined to be a new compound, which was named torvoside W.

Compound **4** (45.7 mg), a white amorphous powder, is dissolved in methanol. The anisaldehyde reagent reaction exhibited a yellow-green color, while the Ehrlich reagent reaction displayed a red color. (+)-HR-ESI-MS analysis revealed an ion peak at *m*/*z* 941.4741 [M + Na]^+^, confirming the molecular formula as C_45_H_74_O_19_ (the calculated value for C_45_H_74_O_19_Na: 941.4722). The ^1^H-NMR (pyridine-*d*_5_, 600 MHz) spectrum exhibited two distinct methyl signals at *δ*_H_ 0.98 (3H, s, H_3_-18) and 0.83 (3H, s, H_3_-19), as well as two doublet methyl signals at *δ*_H_ 1.10 (d, *J* = 6.7 Hz, H_3_-27) and 1.25 (d, *J* = 7.1 Hz, H_3_-21) characteristic of methyl protons in the steroid saponin. The ^13^C-NMR (pyridine-*d*_5_, 150 MHz) spectrum showed 45 carbon signals for compound **4**, including 3 sugar anomeric carbon signals at *δ*_C_ 106.0, 105.6, and 103.6, as well as 4 quaternary carbon signals at *δ*_C_ 37.0, 41.6, 117.6, and 211.1. A comparison with the chemical signals of compound **1** revealed identical sugar unit signals and a relatively low-field *δ*_C_ 117.6 (C-22) signal, indicating the presence of one quinovose, one rhamnose, and one glucose unit, along with a compound featuring a peroxide hydroxyl group configuration at C-22. The *δ*_C_ 211.1 (C-3) carbonyl chemical shift signal and the absence of a *δ*_C_ 70.6 signal indicated that C-3 was a carbonyl group rather than a hydroxyl group. The absolute configuration of C-25 was determined to be *S*, based on the *δ*_H_ 4.17 (H-26a), 3.53 (H-26b), Δab = (0.64 > 0.57 ppm) values. The HMBC spectrum (Figure 2) revealed correlations between *δ*_H_ 4.71 (d, *J* = 7.7 Hz, H-1′) and *δ*_C_ 80.0 (C-6), *δ*_H_ 6.32 (br s, H-1″) and *δ*_C_ 83.8 (C-3′), and *δ*_H_ 4.87 (H-1′″) and *δ*_C_ 75.6 (C-26), elucidating the connection positions of the sugars in compound 4: glucose was connected with C-26, quinovose was connected with C-6, and rhamnose was connected with C-3 of quinovose. Furthermore, the correlation between *δ*_H_ 1.10 (d, *J* = 6.7 Hz, H_3_-27) and *δ*_C_ 28.7 (C-24), 34.9 (C-25), and 75.6 (C-26), the correlation between *δ*_H_ 1.25 (d, *J* = 7.1 Hz, H_3_-21) and *δ*_C_ 64.3 (C-17), 40.4 (C-20), and 117.6 (C-22), the correlation between *δ*_H_ 0.83 (3H, s, H_3_-19) and *δ*_C_ 41.6 (C-13), 56.1 (C-14), and 64.3 (C-17), as well as the correlation between *δ*_H_ 0.98 (3H, s, H_3_-18) and *δ*_C_ 38.9 (C-1), 52.8 (C-5), 53.4 (C-9), and 37.0 (C-10) further supported its structure determination. Therefore, the structure was identified and named torvoside X.

Compound **5** (64 mg), a white amorphous powder, dissolves in methanol. The anisaldehyde reagent and Ehrlich reagent yield positive results, suggesting the presence of a furan steroid compound. (+)-HR-ESI-MS reveals an ion peak at *m*/*z* 885.4866 [M + H]^+^, and combined with NMR spectroscopic data, the molecular formula is determined to be C_45_H_72_O_17_ (calculated as C_45_H_73_O_17_: 885.4848). The ^1^H NMR (pyridine-*d*_5_, 600 MHz) data show two singlet methyl signals at *δ*_H_ 1.03 (3H, s, H_3_-18) and 0.89 (3H, s, H_3_-19), as well as two doublet methyl signals at *δ*_H_ 1.12 (d, *J* = 5.8 Hz, H_3_-27) and 1.72 (d, *J* = 5.6 Hz, H_3_-21), which are characteristic methyl proton signals of steroid glycosides. The ^13^C NMR (pyridine-*d*_5_, 150 MHz) data show that compound **5** has 45 carbon signals, including 3 sugar anomeric carbon signals at *δ*_C_ 103.6, 105.5, and 106.0, as well as a pair of double-bond signals at *δ*_C_ 164.0 (C-22) and 91.6 (C-23). Combined with HSQC spectroscopic data, it is shown that compound **5** has eleven methylene signals at *δ*_C_ 39.0 (C-1), 38.4 (C-2), 40.2 (C-4), 41.1 (C-7), 21.1 (C-11), 39.5 (C-12), 33.6 (C-15), 30.0 (C-24), 75.8 (C-26), 63.0 (C-6′″), and four quaternary carbon signals at *δ*_C_ 211.1 (C-3), 37.1 (C-10), 40.9 (C-13), and 164.0 (C-22). Compound **5** and macaoside J [17] have the same NMR signals, except for the sugar unit part, and both have the same aglycone with double bonds at C-22 and C-23. In the HMBC spectrum (Figure 2), correlations between *δ*_H_ 4.74 (d, *J* = 7.7 Hz, H-1′) and *δ*_C_ 80.2 (C-6), *δ*_H_ 6.32 (br s, H-1″) and *δ*_C_ 83.8 (C-3′), and *δ*_H_ 4.89 (d, *J* = 7.7 Hz, H-1′″) and *δ*_C_ 75.8 (C-26) indicate the connection position of the sugar moiety, with glucose connected to C-26, quinovose connected to C-6, and rhamnose connected to C-3 of quinovose. Moreover, correlations between *δ*_H_ 1.12 (d, *J* = 5.8 Hz, H_3_-27) and *δ*_C_ 30.0 (C-24), 35.2 (C-25), and 75.8 (C-26), *δ*_H_ 1.72 (d, *J* = 5.6 Hz, H_3_-21) and *δ*_C_ 68.1 (C-17), 40.8 (C-20), and 164.0 (C-22), *δ*_H_ 0.89 (3H, s, H_3_-19) and *δ*_C_ 40.9 (C-13), 56.7 (C-14), and 68.1 (C-17), and *δ*_H_ 1.03 (3H, s, H_3_-18) and *δ*_C_ 52.8 (C-5), 53.2 (C-9), and 37.1 (C-10) further confirm its structure. The absolute configuration of C-25 is determined to be the *S* configuration (*δ*_H_ 4.22 (H-26a), 3.54 (H-26b), Δab = 0.68 > 0.57 ppm). Therefore, torvoside Y (**5**) is determined.

Compound **6** (4.3 mg) is a white amorphous powder, which is soluble in methanol. The anisaldehyde reagent reaction shows a yellow-green color, and the Ehrlich reagent reaction shows a red color, indicating that the compound may be a furan steroid compound. (+)-HR-ESI-MS shows an ion peak at *m*/*z* 923.4632[M + Na]^+^, and in combination with NMR spectral data, the molecular formula is confirmed as C_45_H_72_O_18_ (calculated as C_45_H_72_O_18_Na: 923.4616). The ^1^H NMR (pyridine-*d*_5_, 600 MHz) data show three singlet methyl signals at *δ*_H_ 0.99 (3H, s, H_3_-18), 0.82 (3H, s, H_3_-19), and 1.36 (3H, s, H_3_-21), as well as a doublet methyl signal at *δ*_H_ 1.13 (d, *J* = 6.2 Hz, H_3_-27), which are characteristic methyl proton signals for a steroid glycoside. In addition, there are two more high-field doublet methyl signals at *δ*_H_ 1.62 (d, *J* = 5.9 Hz, H_3_-6′) and 1.71 (d, *J* = 6.0 Hz, H_3_-6″). The ^13^C NMR (pyridine-*d*_5_, 150 MHz) data show 45 carbon signals, including 3 sugar anomeric carbon signals at *δ*_C_ 103.6, 105.6, and 106.1, as well as a pair of double-bond signals at *δ*_C_ 157.5 (C-22) and 96.5 (C-23). Combined with HSQC spectral data, it is shown that the compound has 11 methylene signals at *δ*_C_ 38.9 (C-1), 38.4 (C-2), 40.1 (C-4), 41.1 (C-7), 21.1 (C-11), 39.4 (C-12), 33.5 (C-15), 30.0 (C-24), 75.8 (C-26), 63.1 (C-6″′), and 4 quaternary carbon signals at *δ*_C_ 211.1 (C-3), 37.1 (C-10), 40.8 (C-13), and 157.5 (C-22). Compared with compound **5**, it is found that there is only one more oxygenated tertiary methyl signal at *δ*_C_ 82.6, and the signal at *δ*_H_ 1.36 (3H, s, H_3_-21) is a singlet peak, indicating that C-21 is a quaternary carbon signal connected to a hydroxyl group. In the HMBC spectrum (Figure 2), the correlations between *δ*_H_ 4.71 (d, *J* = 7.8 Hz, H_1_-1′) and *δ*_C_ 80.1 (C-6), *δ*_H_ 6.30 (br s, H_1_-1″) and *δ*_C_ 83.8 (C-3′), and *δ*_H_ 4.92 (d, *J* = 7.7 Hz, H_1_-1″′) and *δ*_C_ 75.8 (C-26) indicate the connection positions of the sugar moiety, with glucose connected at C-26, quinovose connected at C-6, and rhamnose connected at the C-3 position of quinovose. Moreover, the correlations between *δ*_H_ 1.13 (d, *J* = 6.2 Hz, H_3_-27) and *δ*_C_ 30.0 (C-24), 35.2 (C-25), and 75.8 (C-26), *δ*_H_ 1.36 (s, H_3_-21) and δC 67.0 (C-17), 82.6 (C-20), and 157.5 (C-22), *δ*_H_ 0.82 (3H, s, H3-19) and *δ*_C_ 40.8 (C-13), 56.5 (C-14), and 67.0 (C-17), as well as *δ*_H_ 0.99 (3H, s, H_3_-18) and *δ*_C_ 38.9 (C-1), 52.8 (C-5), 53.2 (C-9), and 37.1 (C-10) further confirm its structure. The structure of the compound is determined and named torvoside Z.

Sixteen known compounds were identified as torvosides A, H (**7**, **8**) [2], **9** [18], **10** [19], **11**, **13**, **19**, **20** [6], **12**, torvpregnanoside A (**22**) [20], torvoside C, D (**16**, **17**) [5], 26-degluco-torvosides A, H (**15**, **21**) [4], torvoside J (**14**) [21],**18** [4].

#### 2.1.1. Torvoside U (**1**)

White amorphous powder, $\alpha_{D}^{23}$ −59.80 (c 0.1, MeOH); UV (MeOH) λ_max_ 203 (log ε) (0.68) nm; IR (KBr) υ_max_ 3433, 2933, 2878, 1452, 1382, and 579 cm^−1^; ^1^H and ^13^C NMR (pyridine-*d*_5_), see Table 1 and Table 2, HR-ESI-MS *m*/*z* 943.4892 [M + Na]^+^ (C_45_H_76_O_19_), calculated C_45_H_76_O_19_Na: 943.4878.

#### 2.1.2. Torvoside V (**2**)

White amorphous powder, $\alpha_{D}^{22.9}$ −43.11 (c 0.1, MeOH); UV (MeOH) λ_max_ 203.50 (log ε) (1.10) nm; IR (KBr) υ_max_ 3430, 2932, 1383, 1076, and 1042 cm^−1^; ^1^H and ^13^C NMR (pyridine-*d*_5_), see Table 1 and Table 2, HR-ESI-MS *m*/*z* 959.4833 [M + Na]^+^ (C_45_H_76_O_20_), calculated C_45_H_76_O_20_Na: 959.4828.

#### 2.1.3. Torvoside W (**3**)

White amorphous powder, $\alpha_{D}^{23.1}$ −20.00 (c 0.1, MeOH); UV (MeOH) λ_max_ 202.2 (log ε) (3.50) nm; IR (KBr) υ_max_ 3435, 2923, 2852, 1384, and 1075 cm^−1^; ^1^H and ^13^C NMR (pyridine-*d*_5_), see Table 1 and Table 2, HR-ESI-MS *m*/*z* 929.4733 [M + Na]^+^ (C_44_H_74_O_19_), calculated C_44_H_74_O_19_Na: 929.4722.

#### 2.1.4. Torvoside X (**4**)

White amorphous powder, $\alpha_{D}^{23}$ −41.78 (c 0.1, MeOH); UV (MeOH) λ_max_ 203 (log ε) (1.51) nm and 224.50 (log ε) (0.97) nm; IR (KBr) υ_max_ x 3430, 2930, 1383, 1073, and 1051 cm^−1^; ^1^H and ^13^C NMR (pyridine-*d*_5_), see Table 1 and Table 2, HR-ESI-MS *m*/*z* 941.4741 [M + Na]^+^ (C_45_H_74_O_19_), calculated C_45_H_74_O_19_Na: 941.4722.

#### 2.1.5. Torvoside Y (**5**)

White amorphous powder, $\alpha_{D}^{25.7}$ −24.30 (c 0.1, MeOH); UV (MeOH) λ_masx_ 205.6 (log ε) (10.47) nm and 239.20 (log ε) (1.03) nm; IR (KBr) υ_max_ 3435, 2933, 2875, 1382, and 1073 cm^−1^; ^1^H and ^13^C NMR (pyridine-*d*_5_), see Table 1 and Table 2, HR-ESI-MS *m*/*z* 885.4866 [M + H]^+^ (C_45_H_72_O_17_), calculated C_45_H_73_O_17_: 885.4848.

#### 2.1.6. Torvoside Z (**6**)

White amorphous powder, $\alpha_{D}^{25.8}$ −15.49 (c 0.1, MeOH); UV (MeOH) λ_max_ 206 (log ε) (10.15) nm and 233.50 (log ε) (2.38) nm; IR (KBr) υ_max_ 3411, 2934, 1384, and 1077 cm^−1^; ^1^H and ^13^C NMR (pyridine-*d*_5_), see Table 1 and Table 2, HR-ESI-MS *m*/*z* 923.4632 [M + Na]^+^ (C_45_H_72_O_18_), calculated C_45_H_72_O_18_Na: 923.4616.

### 2.2. Biological Activity Results

#### 2.2.1. Screening of Anti-Epileptic Activity of the Compounds

**Figure 4:**
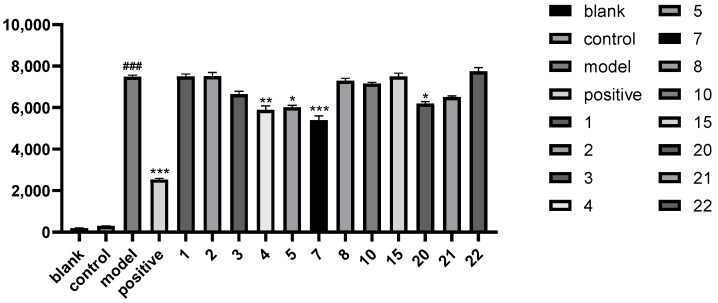
The anti-epileptic activity of compounds against the PTZ-induced seizure model in zebrafish (### *p* < 0.001 vs. control, * *p* < 0.05 vs. model, ** *p* < 0.01 vs. model, *** *p* < 0.001 vs. model).

The present study successfully established a zebrafish epilepsy model induced by pentylenetetrazole (PTZ). Throughout the experiment, the concentration of dimethyl sulfoxide (DMSO) was maintained at 0.1%, and the final concentration for optimal induction was 500 μM PTZ. In the positive drug experiment with phenytoin sodium, it was observed that increasing the concentration up to 600 μM significantly reduced the locomotor activity of zebrafish larvae, but further increasing the concentration did not result in additional suppression of movement. Compounds **1**–**5**, **7**–**8**, **10**, **15**, **20**–**22** were utilized in the experiment. The results demonstrated that the steroidal saponins torvoside X (**4**), torvoside Y (**5**), torvoside A (**7**), and (25*S*)-3-oxo-5*α*-spirostan-6*α*-yl-*O*-*β*-d-xylopyranoside (**20**) exhibited anti-epileptic activity (Figure 4). Active compounds with a keto group at position C-3 and furan steroid saponins (**4**, **5**, **7**, **20**) showed better activity.

#### 2.2.2. Screening for Anti-Liver-Cancer Activity

**Figure 5:**
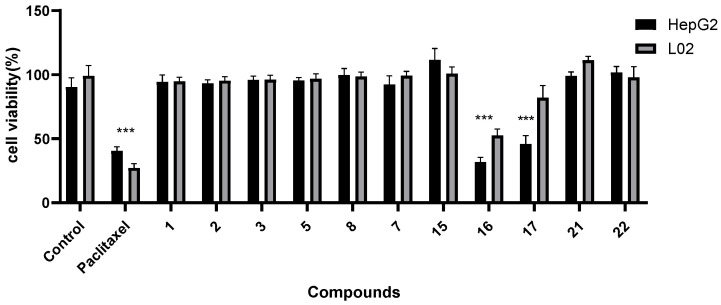
Cell viability of compounds in HepG2 and L02 (*** *p* < 0.001 vs. model).

The experimental results demonstrated that the compounds showed no liver toxicity toward HepG 2 cells and normal L02 cells, except for compounds **16** and **17**, as determined at a concentration of 70 μM using the MTT assay. This suggests that the aqueous extract of water eggplant contains several steroidal saponins with minimal liver toxicity, making it suitable for consumption (Figure 5).

## 3. Discussion

Steroidal saponins, found extensively in plants, have over ten thousand known variants. Their pharmacological activity has garnered global interest. These saponins exhibit significant variance in structure and properties, depending on their source. Furthermore, steroidal saponins from different species of the same plant can display distinct pharmacological activities [14].

In this study, a total of 22 compounds were isolated and purified from the fruit of *Solanum torvum* using a series of sophisticated separation techniques. These compounds primarily consist of steroidal glycosides with sugar substitutions at the C6 position. Compounds **1**–**6** are novel furostanol glycosides, characterized by dehydration of the hydroxy group at C22, resulting in formation of a double bond at either position 20,22 or 22,23, and in some cases, the formation of peroxide derivatives. Based on the structural characteristics of these compounds, it can be inferred that they are generated as natural products undergo boiling. The C22 position, which exhibits a hemiacetal structure, is inherently unstable and prone to oxidation under normal heating conditions, giving rise to this unique class of compounds isolated in this study. Furthermore, during the isolation process, the hydroxy group at the C22 position of the compounds may undergo methoxylation when exposed to methanol. To prevent such occurrences, a 50% acetonitrile–water solution is commonly employed to revert the hydroxy group back to its original state.

A screening of compounds from water eggplant for anti-epileptic activity revealed that steroidal glycosides (**4**, **5**, **7**, **20**) with C-6-linked quinovose and rhamnose, a hydroxy group at C-22, a peroxide hydroxy group, or double bonds at C22 and C23 exhibited remarkable anti-epileptic properties. Through the evaluation of hepatotoxicity, spirostanol glycosides (**16**, **17**) with a hydroxy group at position 23 on the F ring were found to possess anti-liver-cancer activity, and most compounds showed no hepatotoxicity. Some researchers have delved deeper into the structure–activity relationship of steroidal saponins. They found that rhamnose [22] is the crucial sugar unit for pharmacological effects, and the 6-OH [23] compound is also a significant therapeutic site. According to the findings of this study and a review of the literature, most compounds isolated from *Solanum torvum* contain rhamnose. The 6-OH is replaced by the sugar unit, resulting in different effects. Therefore, this study suggests new possibilities for the active site of steroidal saponins based on these findings.

## 4. Materials and Methods

### 4.1. Equipment 

NMR analyses were carried out on a Bruker 600 MHz spectrometer (Bruker BioSpin GmbH, Ettlingen, Germany). HR-ESI-MS data was recorded using an Agilent 6500 Q-TOF spectrometer (Agilent Technologies, Santa Clara, CA, USA). HPLC-UV analyses were performed on a Waters 2695 series system (Waters Corporation, Milford, MA, USA) with an Agilent-SB-C18 column (250 × 4.6 mm, 5 μm). For semi-preparative HPLC, a Newstyle^®^ laboratory liquid chromatography system (Jiangsu Hanbang Technology, Huaian, China) equipped with an NU-3000 detector and two NP-7000C infusion units was used, employing YMC-Pack ODS-A C18 column (250 mm × 10 mm, 5 μm) (YMC, Devens, MA, USA) and COSMOSIL Cholester column (COSMOSIL, Tokyo, Japan, 150 mm × 10 mm, 5 μm). TLC was performed using GF254 plates and HF-254 plates (Qingdao Ocean Chemical Co., Ltd., Qingdao, China). A zebrafish behavior trajectory tracker (Noldus Information Technology, Sheffield, UK) and a microplate reader (Tecan Trading AG, Männedorf, Switzerland) were utilized for biological activity analysis and data acquisition, respectively.

### 4.2. Chemicals and Reagents

E3 Water (60×): NaCl 34.8g, KCl 1.6g, CaCl_2_·2H_2_O 5.8 g, MgCl_2_·6H_2_O 9.78 g, with a final volume of 2L. This solution is adjusted to a pH value within the range of 6.9–7.2 and a conductivity level between 480 and 510 μs/cm prior to high-pressure sterilization. Subsequently, it is diluted to a 1 × concentration using reverse osmosis water for zebrafish culture. CaCl_2_.2H_2_O, NaCl, KCl, and MgCl_2_.6H_2_O were obtained from Tianjin Fengchuan Chemical Reagent Company (Tianjin, China); dimethyl sulfoxide (DMSO), pentylenetetrazole (PTZ), and phenytoin sodium were obtained from Tianjin Zhiyuan Chemical Reagent Company (Tianjin, China); the 48-well plate, pipette gun, and constant temperature incubator were acquired from Shanghai Yiheng Scientific Instrument Company (Shanghai, China); DEME Medium was sourced from Dalian Meilun Biotechnology Co., Ltd., Dalian, China; FBS was obtained from Thermo Fisher Company, Waltham, MA, USA; streptomycin and penicillin were acquired from Cytiva Company, Marlborough, MA, USA; 3-(4,5-dimethylthiazol-2-yl)-2,5-diphenyltetrazolium bromide (MTT) was obtained from Solarbio Science & Technology Co., Ltd., Beijing, China. HepG2 cells were obtained from the China Center for Type Culture Collection (CCTCC, Wuhan, China), and L02 cells were graciously provided by Southern Medical University (Beijing, China).

### 4.3. Plant Material

Fresh fruits of *Solanum torvum* were harvested between September and October 2017 in the Dehong Dai and Jingpo Autonomous Prefecture of Yunnan Province, China. This variety is known locally as “little bitter fruit” and was identified by Professor Dr. Hai-zhou Li. from the School of Life Science and Technology of Kunming University of Science and Technology.

### 4.4. Extraction and Isolation

Three kilograms of dried water eggplant was pulverized and extracted four times with 70% methanol/water at ambient temperature, resulting in four hundred thirty-five grams of concentrated extract. The extract was subsequently subjected to sequential extraction three times using petroleum ether and dichloromethane. The resulting fractions were concentrated under reduced pressure, yielding the petroleum ether phase (27 g), dichloromethane phase (94 g), and aqueous phase (250 g). A total of 22 compounds were isolated from the aqueous phase utilizing silica gel column chromatography, ODS reverse phase column chromatography, and semi-preparative techniques. Among these compounds, compounds **1**–**6** were newly identified.

### 4.5. Bioactivity Assays

#### 4.5.1. Anti-Epileptic Activity Studies

The experiment was conducted using 7-day-old zebrafish larvae. Each well of a 48-well plate contained one fish, and each group was replicated 7 times. The zebrafish larvae were observed for their locomotor behavior in a dark environment using a zebrafish behavior tracking analyzer. After a stable period of 10 min, the distance traveled by the larvae within 1 h, with a speed exceeding 20 mm/s, was recorded. The experiment involved testing different concentrations of DMSO, pentylenetetrazole (PTZ), and the positive drug phenytoin sodium. The blank group was treated with 400 μL of E3 water. The effects of different concentrations of DMSO (0.1%, 1%, 3%) were compared, and it was observed that a concentration of 0.1% DMSO had no impact on zebrafish locomotor behavior. In the model group, 380 μL of E3 water was added and pre-incubated for 1 h at 28 °C, followed by the addition of 20 μL of PTZ solution at different concentrations (1.25 mM, 2.5 mM, 5 mM, 10 mM, 20 mM). The results demonstrated that the addition of 20 μL of 10 mM PTZ (final concentration 500 μM) significantly induced rapid locomotion in zebrafish. Therefore, it was determined that a final concentration of 500 μM PTZ is the optimal induction concentration for the zebrafish PTZ-induced seizure model. In the dosing experimental group, after pre-treatment with different concentrations of phenytoin sodium solution (200 μM, 300 μM, 400 μM, 500 μM, 600 μM, 800 μM) for 1 h at 28 °C, followed by the addition of 20 μL of 10 mM PTZ solution, it was observed that phenytoin sodium significantly reduced zebrafish seizure behavior when the concentration exceeded 600 μM.

The primary compounds **1**–**5**, **7**–**8**, **10**, **15**, **20**–**22** isolated from the fruit of *S. torvum* were examined for their anti-epileptic activity. The experiment comprised distinct groups: a blank group, a solvent control group, a model group, a positive drug group, and a compound group. In the blank group, 400 μL of E3 water was administered, while the solvent control group received 400 μL of 0.1% DMSO. The model group was given 380 μL of E3 water; the positive drug group received 380 μL of 600 μM phenytoin sodium solution; and the compound group received 380 μL of 70 μM monomer compound. After a pre-incubation period at 28 °C for 1 h, 20 μL of 10 mM PTZ solution was added. The movement distance of larvae traveling at a speed exceeding 20 mm/s within 1 h was measured in a dark environment.

#### 4.5.2. Screening for Hepatotoxicity

Cytotoxic activity was evaluated using the MTT assay. HepG2 cells were detached using trypsin during the logarithmic growth phase and seeded into a 96-well plate at a density of 10,000 cells/mL. Each well received 100 μL of cell suspension (containing 1000 cells per well) and was then incubated at 37 °C in a 5% CO_2_ incubator for 24 h. After pre-culture, the original culture medium was removed and replaced with 200 μL of the medium containing the respective drugs in each well. In addition, solvent control and positive control groups were also established, with 5 replicate wells per group. The 96-well plate was then incubated at 37 °C in a 5% CO_2_ incubator for 72 h. Subsequently, 20 μL of MTT solution was added to each well and incubated at 37 °C for 4 h. Absorbance at 490 nm was measured using a microplate reader (*n* = 5). The optical density value for each well represents cell viability/proliferation. The same steps were followed for the L02 cell experiment.

### 4.6. Statistical Analysis

The results were expressed as mean ± standard error of the mean (SEM) and subjected to analysis of variance (ANOVA) with post hoc Tukey and Dunnett tests—with 5% significance level—using the GraphPad Prism 5 Demo https://www.graphpad-prism.cn/, accessed on 3 March 2024

### 4.7. Animal Ethics

This study was approved by the animal ethics committee (Kunming University of Science and Technology Experimental Animal Ethics Committee KMUST2023SK04001, approval data is 1 April 2023).

## 5. Conclusions

In this study, twenty-two steroidal saponins were isolated and purified from the fruits grown in Yunnan, China, including six new compounds: torvosides U–Z (**1**–**6**). During drying and cooking, the saponins may undergo transformation, resulting in small amounts of sapogenins. These transformations can include dehydration of hydroxyl groups at position C22, formation of double bonds at position 20, 22 or 22, 23, and even formation of peroxide products. The saponin compounds torvoside X (**4**), torvoside Y (**5**), torvoside A (**7**), and (25*S*)-3-oxo-5*α*-spirostan-6*α*-yl-*O*-*β*-d-xylopyranoside (**20**), which are glycosylated at C-6, exhibited certain anti-epileptic activity in the pentylenetetrazole-induced zebrafish seizure model. No antiproliferative activity was detected when tested on the cancer cell line HepG2, and no hepatotoxic effect was noted on normal liver cell line LO2. Functional foods have rapidly become significant in people’s everyday routines due to the growing focus on health care. Both medicinal and edible plants now play a crucial role in production, daily life, and scientific research. These discoveries affirm the traditional Chinese practice of consuming water eggplants, providing a solid basis for the comprehensive use of this medicinal and culinary plant resource.

## Figures and Tables

**Table 1:** ^1^H-NMR data (600 MHz, C_5_D_5_N) of compounds **1**–**6** (δ in ppm, J in Hz).

No.	1	2	3	4	5	6
1	0.88 m	0.88 m	1.16 m	0.91 m	1.15 m	1.14 m
	1.59 m ^a^	1.58 m ^a^	1.77 m	1.60 m ^a^	1.77 m	1.77 m
2	1.34 m	1.24 m	2.27 m	1.37 m	2.29 m	2.28 m
	1.90 m	1.80 m	2.41 m	1.91 m	2.39 m	2.39 m
3	3.70 m ^a^	3.72 m	/	3.81 m	/	/
4	1.64 m	1.66 m	2.44 m	1.72 m	2.44 m	2.45 m
	3.18 dd (14.5, 15.0)	3.21 m	3.55 m	3.25 dd (14.1, 14.5)	3.56 m ^a^	3.58 m
5	1.26 m	1.25 m	1.57 m ^a^	1.39 m	1.59 m ^a^	1.58 m ^a^
6	3.66 m	3.70 m	3.69 m	3.74 m ^a^	3.74 m	3.70 m
7	1.11 m	1.06 m	1.14 m	1.17 m	1.16 m	1.08 m
	2.45 m	2.45 m	2.44 m	2.43 m ^a^	2.45 m	2.42 m
8	1.60 m ^a^	1.51 m ^a^	1.62 m	1.58 m	1.60 m ^a^	1.59 m ^a^
9	0.54 m	0.53 m	0.56 m	0.57 m	0.56 m	0.51 m
10	/	/	/	/	/	/
11	1.17 dd (3.9, 12.2)	1.17 dd (3.7, 4.2)	1.27 dd (7.2, 8.0)	1.22 dd (5.8, 6.1)	1.25 m	1.24 m
	1.41 m	1.41 m	1.39 m	1.43 m	1.39 m	1.39 m
12	0.98 br d (10.4)	0.99 d (4.6)	1.02 d (8.1)	1.01 m	1.11 d (6.2)	1.13 d (6.6)
	1.65 m	1.64 m	1.66 m	1.65 m	1.78 m	1.74 m
13	/	/	/	/	/	/
14	0.95 m	0.86 m	0.94 m	0.94 m	0.93 m	0.87 m
15	1.71 m	1.75 m	1.39 m	1.77 m ^a^	1.45 m	1.37 m
	2.02 m	2.03 m ^a^	1.90 m	2.05 m	2.05 m	1.98 m
16	4.77 m	4.73 m	4.81 m	4.80 m	5.18 m	4.90 m ^a^
17	1.80 m	1.76 m	1.81 m	1.80 m	2.22 m	2.03 m
18	0.81 s	0.82 s	0.98 s	0.81 s	1.03 s	0.99 s
19	0.80 s	0.77 s	0.83 s	0.80 s	0.89 s	0.82 s
20	2.39 m	2.37 m ^a^	2.40 m	2.41 m ^a^	2.43 m	/
21	1.25 d (6.0)	1.23 d (7.0)	1.25 d (7.1)	1.25 d (6.8)	1.72 d (5.6)	1.36 s
22	/	/	/	/	/	/
23	2.24 m	2.22 m	1.31 m	1.26 m	4.55 t (6.9)	4.34 m
	2.33 m	2.31 m ^a^	1.28 m	1.32 m		
24	1.57 m	1.55 m	1.58 m	1.57 m ^a^	2.14 m	2.20 m
	2.04 m	2.04 m ^a^	2.05 m	2.04 m	2.56 m	2.52 m
25	1.96 m	1.96 m	1.96 m	1.96 m	2.15 m	2.14 m
26	3.53 m	3.52 m	3.53 m	3.51 m	3.54 m ^a^	3.58 m
	4.16 m	4.15 m	4.17 m	4.16 m	4.22 m	4.21 m
27	1.10 d (5.6)	1.10 d (6.5)	1.10 d (6.7)	1.10 d (6.5)	1.12 d (5.8)	1.13 d (6.2)
	6-*O*-Qui	6-*O*-Glc	6-*O*-Qui	6-*O*-Qui	6-*O*-Qui	6-*O*-Qui
1′	4.80 d (6.0)	4.85 d (6.0)	4.71 d (7.7)	4.86 d (7.1)	4.74 d (7.7)	4.71 d (7.8)
2′	4.04 dd (3.9, 4.2)	4.07 m	4.02 m	4.08 m	4.03 dd (8.7, 8.9)	4.01 m
3′	4.29 t (9.0) ^a^	4.41 m	4.28 m ^a^	4.12 m	4.28 m ^a^	4.26 m ^a^
4′	3.62 t (4.6)	5.16 m	3.64 t (9.4)	3.64 t (9.1)	3.62 t (9.5)	3.64 m
5′	4.64 m	3.90 dd (9.2, 9.5)	4.65 m	4.64 m	4.64 m	4.62 m
6′	1.73 d (6.0)	4.58 m	1.65 d (6.0)	1.60 d (5.8) ^a^	1.64 d (6.1)	1.62 d (5.9)
		4.48 m				
	Rha	Rha	Rha	Xyl	Rha	Rha
1″	6.36 br s	6.39 br s	6.32 br s	5.30 d (7.4)	6.32 br s	6.30 br s
2″	3.76 m ^a^	4.65 m	3.76 m	4.29 m	3.76 m	3.74 m
3″	4.85 dd (4.5, 4.8)	4.87 dd (7.8, 8.0)	4.86 dd (3.9, 9.0)	4.22 m ^a^	4.87 dd (7.8, 8.0)	4.85 m
4″	4.39 t (4.8)	4.39 t (5.0)	4.40 t (9.5)	4.22 m ^a^	4.38 t (9.3)	4.38 t (9.6)
5″	5.07 m	5.06 m	5.05 m	3.77 m	5.06 m	5.03 m
				4.34 m		
6″	1.64 d (6.2)	1.72 d (6.5)	1.72 d (6.2)		1.73 d (5.6) ^a^	1.71 d (6.0)
	26-*O*-Glc	26-*O*-Glc	26-*O*-Glc	26-*O*-Glc	26-*O*-Glc	26-*O*-Glc
1‴	4.86 d (7.6)	4.88 d (7.7)	4.87 d (7.7)	4.87 d (7.6)	4.89 d (7.7)	4.92 d (7.7)
2‴	4.06 m	4.08 m	4.08 m	4.10 m	4.09 m	4.09 m
3‴	4.29 m ^a^	4.28 m ^a^	4.28 m ^a^	4.29 m ^a^	4.29 m ^a^	4.28 m ^a^
4‴	4.29 m ^a^	4.30 m ^a^	4.29 m ^a^	4.30 m ^a^	4.31 m ^a^	4.29 m ^a^
5‴	3.98 m	3.98 m	3.99 m	3.99 m	3.98 m	3.98 m
6‴	4.58 dd (4.6, 11.6)	4.60 dd (4.0, 11.7)	4.59 dd (4.0, 9.4)	4.44 dd (4.9, 11.8)	4.40 dd (9.7, 11.3)	4.43 dd (6.0, 11.6)
	4.44 dd (5.9, 11.6)	4.43 dd (4.8, 11.7)	4.41 dd (5.4, 9.4)	4.59 dd (4.5, 11.8)	4.59 dd (9.4, 11.3)	4.58 dd (5.1, 11.6)

^a^: Overlapped signals, s: singlet, d: doublet, t: triplet, m: multiplet, br s: broad singlet, br d: broad doublet, ′: the first sugar, ″: the second sugar, ‴: the third sugar, /: no hydrogen.

**Table 2:** ^13^C-NMR data (150 MHz, C_5_D_5_N) of compounds **1**–**6** (δ in ppm).

No.	1	2	3	4	5	6
1	38.0 (CH_2_)	38.0 (CH_2_)	38.9 (CH_2_)	38.0 (CH_2_)	39.0 (CH_2_)	38.9 (CH_2_)
2	32.7 (CH_2_)	32.6 (CH_2_)	38.4 (CH_2_)	32.6 (CH_2_)	38.4 (CH_2_)	38.4 (CH_2_)
3	70.9 (CH)	70.9 (CH)	211.1 (C)	70.9 (CH)	211.1 (C)	211.1 (C)
4	33.5 (CH_2_)	33.5 (CH_2_)	39.9 (CH_2_)	33.6 (CH_2_)	40.2 (CH_2_)	40.1 (CH_2_)
5	51.7 (C)	51.7 (C)	52.8 (C)	51.7 (C)	52.8 (C)	52.8 (C)
6	79.4 (CH)	79.8 (CH)	80.0 (CH)	79.4 (CH)	80.2 (CH)	80.1 (CH)
7	41.7 (CH_2_)	41.6 (CH_2_)	41.3 (CH_2_)	41.7 (CH_2_)	41.1 (CH_2_)	41.1 (CH_2_)
8	34.4 (CH)	34.3 (CH)	34.2 (CH)	34.4 (CH)	33.7 (CH)	33.6 (CH)
9	54.1 (CH)	54.1 (CH)	53.4 (CH)	54.1 (CH)	53.2 (CH)	53.2 (CH)
10	37.0 (C)	37.0 (C)	37.0 (C)	37.0 (C)	37.1 (C)	37.1 (C)
11	21.5 (CH_2_)	21.4 (CH_2_)	21.5 (CH_2_)	21.5 (CH_2_)	21.1 (CH_2_)	21.1 (CH_2_)
12	40.1 (CH_2_)	40.1 (CH_2_)	40.1 (CH_2_)	40.1 (CH_2_)	39.5 (CH_2_)	39.4 (CH_2_)
13	41.6 (C)	41.6 (C)	41.6 (C)	41.6 (C)	40.9 (C)	40.8 (C)
14	56.4 (CH)	56.4 (CH)	56.1 (CH)	56.3 (CH)	56.7 (CH)	56.5 (CH)
15	32.5 (CH_2_)	32.5 (CH_2_)	32.6 (CH_2_)	32.5 (CH_2_)	33.6 (CH_2_)	33.5 (CH_2_)
16	82.6 (CH)	82.6 (CH)	82.6 (CH)	82.6 (CH)	84.4 (CH)	84.1 (CH)
17	64.1 (CH)	64.3 (CH)	64.3 (CH)	64.4 (CH)	68.1 (CH)	67.0 (CH)
18	13.9 (CH_3_)	13.8 (CH_3_)	12.8 (CH_3_)	13.9 (CH_3_)	12.9 (CH_3_)	12.8 (CH_3_)
19	16.7 (CH_3_)	16.7 (CH_3_)	16.7 (CH_3_)	16.7 (CH_3_)	14.0 (CH_3_)	14.1 (CH_3_)
20	40.4 (CH)	40.4 (CH)	40.4 (CH)	40.4 (CH)	40.8 (CH)	82.6 (C)
21	16.2 (CH_3_)	16.2 (CH_3_)	16.2 (CH_3_)	16.2 (CH_3_)	22.2 (CH_3_)	15.5 (CH_3_)
22	117.6 (C)	117.6 (C)	117.6 (C)	117.6 (C)	164.0 (C)	157.5 (C)
23	32.3 (CH_2_)	32.2 (CH_2_)	30.3 (CH_2_)	30.3 (CH_2_)	91.6 (CH)	96.5 (CH)
24	28.7 (CH_2_)	28.7 (CH_2_)	28.7 (CH_2_)	28.7 (CH_2_)	30.0 (CH_2_)	30.0 (CH_2_)
25	34.9 (CH)	34.9 (CH)	34.9 (CH)	34.9 (CH)	35.2 (CH)	35.2 (CH)
26	75.6 (CH_2_)	75.6 (CH_2_)	75.6 (CH_2_)	75.6 (CH_2_)	75.8 (CH_2_)	75.8 (CH_2_)
27	17.9 (CH_3_)	17.9 (CH_3_)	17.9 (CH_3_)	17.9 (CH_3_)	17.9 (CH_3_)	17.9 (CH_3_)
1′	105.9(CH)	106.2 (CH)	105.9 (CH)	105.6 (CH)	106.0 (CH)	106.1 (CH)
2′	76.7 (CH)	76.6 (CH)	76.4 (CH)	75.1 (CH)	77.0 (CH)	76.4 (CH)
3′	83.3 (CH)	83.5 (CH)	83.8 (CH)	87.9 (CH)	83.8(CH)	83.8 (CH)
4′	75.6 (CH)	70.2 (CH)	75.5 (CH)	75.3 (CH)	76.3 (CH)	75.5 (CH)
5′	73.0 (CH)	78.4 (CH)	73.1 (CH)	72.7 (CH)	74.5 (CH)	73.1 (CH)
6′	19.1 (CH_3_)	62.8 (CH_2_)	19.0 (CH_3_)	19.0 (CH_3_)	19.0 (CH_3_)	19.2 (CH_3_)
1″	103.4 (CH)	103.3 (CH)	103.6 (CH)	106.9 (CH)	103.6 (CH)	103.6 (CH)
2″	73.1 (CH)	73.1 (CH)	73.1 (CH)	75.6 (CH)	73.1 (CH)	73.1 (CH)
3″	73.0 (CH)	73.0 (CH)	72.9 (CH)	78.7 (CH)	72.9 (CH)	72.9 (CH)
4″	74.5 (CH)	74.5 (CH)	74.5 (CH)	71.3 (CH)	75.5 (CH)	74.5 (CH)
5″	70.2 (CH)	69.9 (CH)	70.3 (CH)	67.8 (CH_2_)	70.3 (CH)	70.3 (CH)
6″	19.1 (CH_3_)	19.1 (CH_3_)	19.1 (CH_3_)	/	19.2 (CH_3_)	19.0 (CH_3_)
1‴	105.6 (CH)	105.6 (CH)	105.6 (CH)	105.5 (CH)	105.5 (CH)	105.6(CH)
2‴	75.5 (CH)	75.6 (CH)	75.6 (CH)	75.8 (CH)	75.6 (CH)	75.7 (CH)
3‴	78.9 (CH)	78.9 (CH)	78.9 (CH)	78.9 (CH)	78.9 (CH)	78.9 (CH)
4‴	71.9 (CH)	71.9 (CH)	71.9 (CH)	71.9 (CH)	71.9 (CH)	71.9 (CH)
5‴	78.9 (CH)	78.9 (CH)	78.9 (CH)	78.9 (CH)	79.0 (CH)	79.0 (CH)
6‴	63.1 (CH_2_)	63.1 (CH_2_)	63.1 (CH_2_)	63.1 (CH_2_)	63.0 (CH_2_)	63.1 (CH_2_)

## Data Availability

Data are contained within the article and Supplementary Materials.

## Supplementary Materials

The following supporting information can be downloaded at: https://www.mdpi.com/article/10.3390/molecules29061316/s1. Figures S1–S50: Key HMBC, HSQC, HRESI, CD, IR, and NMR spectra of compounds **1**–**6**.
