# Supplementary material for: Steroidal Saponins from Water Eggplant (Fruits of Solanum torvum) Exhibit Anti-Epileptic Activity against Pentylenetetrazole-Induced Seizure Model in Zebrafish
Source: Molecules. 2024 Mar 15;29(6):1316. doi: 10.3390/molecules29061316 (PMC10974013; doi:10.3390/molecules29061316)

Supplementary data

Steroidal Saponins From Water Rggplant (the Fruits of *Solanum torvum*) Exhibit Antiepileptic Activity Against the Pentylenetetrazole Induced Seizures Model in Zebrafish

Rui Ren, Ming-yan Zhang, Teng-yun Shu, Ya-ting Kong, Li-hua Su*, Hai-zhou Li*

Faculty of Life Science and Technology, Kunming University of Science and Technology,

Kunming 650500, People's Republic of China

Corresponding author. Tel: 86-871-65920253

E-mail address: sulh@kust.edu.cn (Su L.H.), lihaizhou@kust.edu.cn (Li H.Z.);

List of Contents

Figure S1. HR-ESI-MS Spectrum of compound **1** (1)
Figure S2. UV spectrum of compound **1** (1)
Figure S3. IR spectrum of compound **1** (2)
Figure S4. Optical rotations spectrum of compound **1** (2)
Figure S5. ^{1}H-NMR spectrum of compound **1** (3)
Figure S6. ^{13}C-NMR spectrum of compound **1** (3)
Figure S7. HSQC spectrum of compound **1** (4)
Figure S8. HMBC spectrum of compound **1** (4)
Figure S9. ^{1}H-^{1}H COSY spectrum of compound **1** (5)
Figure S10. ROESY spectrum of compound **1** (5)
Figure S11. HR-ESI-MS Spectrum of compound **2** (6)
Figure S12. UV spectrum of compound **2** (6)
Figure S13. IR spectrum of compound **2** (7)
Figure S14. Optical rotations spectrum of compound **2** (7)
Figure S15. ^{1}H-NMR spectrum of compound **2** (8)
Figure S16. ^{13}C-NMR spectrum of compound **2** (8)
Figure S17. HSQC spectrum of compound **2** (9)
Figure S18. HMBC spectrum of compound **2** (9)
Figure S19. HR-ESI-MS Spectrum of compound **3** (10)
Figure S20. UV spectrum of compound **3** (10)
Figure S21. IR spectrum of compound **3** (11)
Figure S22. Optical rotations spectrum of compound **3** (11)
Figure S23. ^{1}H-NMR spectrum of compound **3** (12)
Figure S24. ^{13}C-NMR spectrum of compound **3** (12)
Figure S25. HSQC spectrum of compound **3** (13)
Figure S26. HMBC spectrum of compound **3** (13)
Figure S27. HR-ESI-MS Spectrum of compound **4** (14)
Figure S28. UV spectrum of compound **4** (14)
Figure S29. IR spectrum of compound **4** (15)
Figure S30. Optical rotations spectrum of compound **4** (15)
Figure S31. ^{1}H-NMR spectrum of compound **4** (16)
Figure S32. ^{13}C-NMR spectrum of compound **4** (16)
Figure S33. HSQC spectrum of compound **4** (17)
Figure S34. HMBC spectrum of compound **4** (17)
Figure S35. HR-ESI-MS Spectrum of compound **5** (18)
Figure S36. UV Spectrum of compound **5** (18)
Figure S37. IR spectrum of compound **5** (19)
Figure S38. Optical rotations spectrum of compound **5** (19)
Figure S39. ^{1}H-NMR spectrum of compound **5** (20)
Figure S40. ^{13}C-NMR spectrum of compound **5** (20)
Figure S41. HSQC spectrum of compound **5** (21)
Figure S42. HMBC spectrum of compound **5** (21)

Figure S43. HR-ESI-MS Spectrum of compound **6** (22)
Figure S44. UV spectrum of compound **6** (22)
Figure S45. IR spectrum of compound **6** (23)
Figure S46. Optical rotations spectrum of compound **6** (23)
Figure S47. ^{1}H-NMR spectrum of compound **6** (24)
Figure S48. ^{13}C-NMR spectrum of compound **6** (24)
Figure S49. HSQC spectrum of compound **6** (25)
Figure S50. HMBC spectrum of compound **6** (25)

Figure S1. HR-ESI-MS Spectrum of compound **1**

+ Scan (rt: 0.077-0.042min, 23 scans) Frag=175.0V Substract

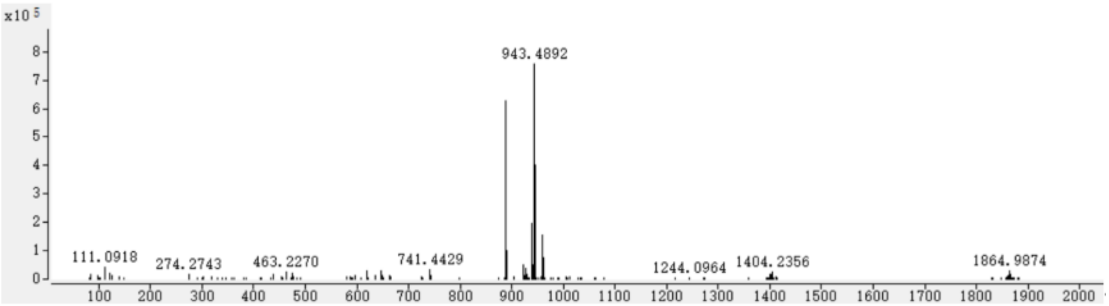

Counts vs. Mass-to-Charge (m/z)

Figure S2. UV spectrum of compound **1**

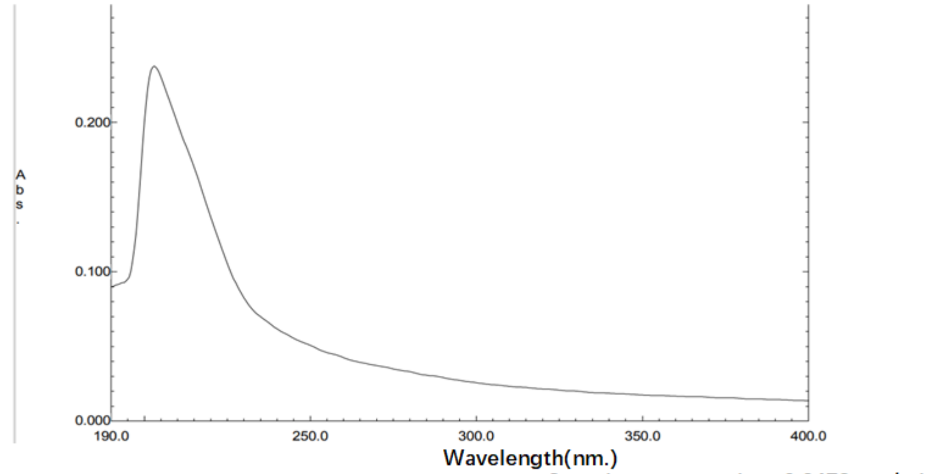

Created in:	18:31	2023/6/8	Sample concentration	0.3478 mg/ml
data:	primeval		solvent:	methanol

Measurement mode: Abs.
Scanning speed: Medium speed
Slit: 5
Sampling interval: 0.5

	Wavelength(nm.)	Abs.
1	203	0.2375

Figure S3. IR spectrum of compound **1**

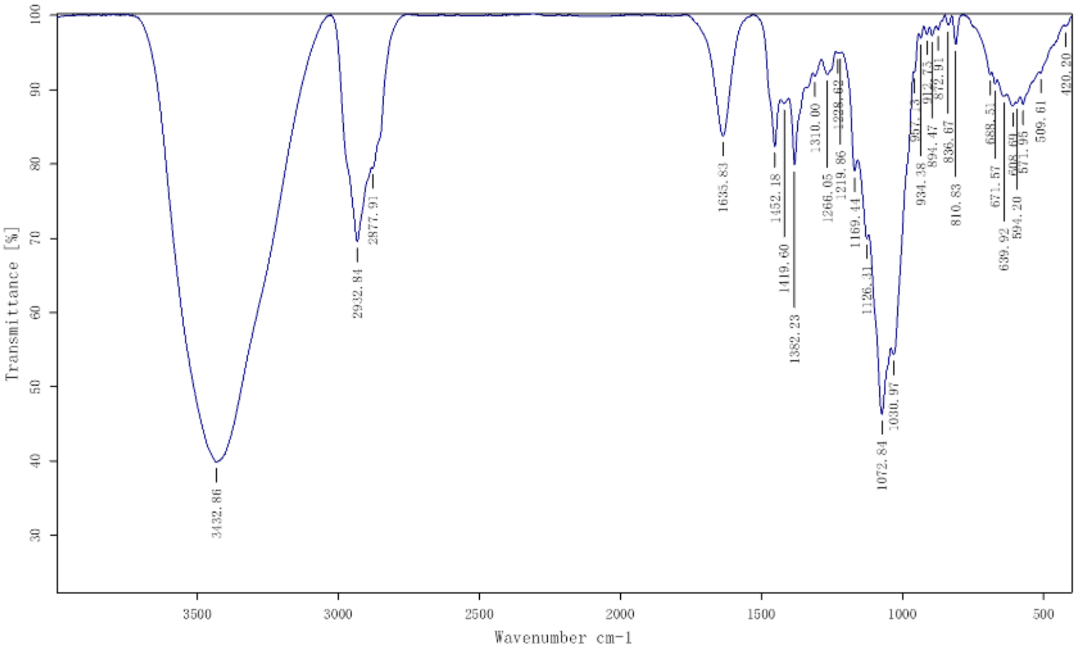

Figure S4. Optical rotations spectrum of compound **1**

Rudolph Research Analytical

This sample was measured on an Autopol VI, Serial #91058
Manufactured by Rudolph Research Analytical, Hackettstown, NJ, USA.

Measurement Date : Thursday, 14-OCT-2021

Set Temperature : OFF

Time Delay : Disabled

Delay between Measurement : Disabled

n	Average	Std.Dev.	% RSD	Maximum	Minimum
5	-59.80	0.45	-0.75	-59.00	-60.00

S.No	Sample ID	Time	Result	Scale	OR °Arc	WLG.nm	Lg.mm	Conc.g/100ml	Temp.
1	zmy-1	04:29:57 PM	-60.00	SR	-0.060	589	100.00	0.100	23.0
2	zmy-1	04:30:04 PM	-59.00	SR	-0.059	589	100.00	0.100	23.0
3	zmy-1	04:30:11 PM	-60.00	SR	-0.060	589	100.00	0.100	23.0
4	zmy-1	04:30:17 PM	-60.00	SR	-0.060	589	100.00	0.100	23.0
5	zmy-1	04:30:24 PM	-60.00	SR	-0.060	589	100.00	0.100	23.0

Figure S5. ^{1}H-NMR spectrum of compound **1**

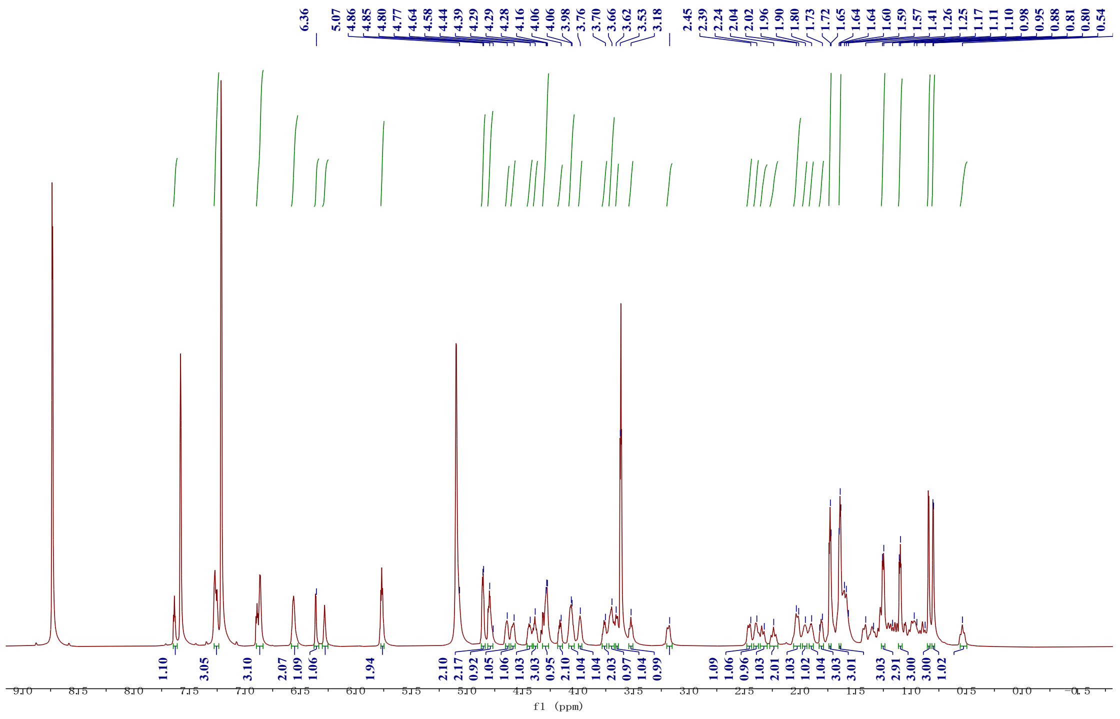

Figure S6. ^{13}C-NMR spectrum of compound **1**

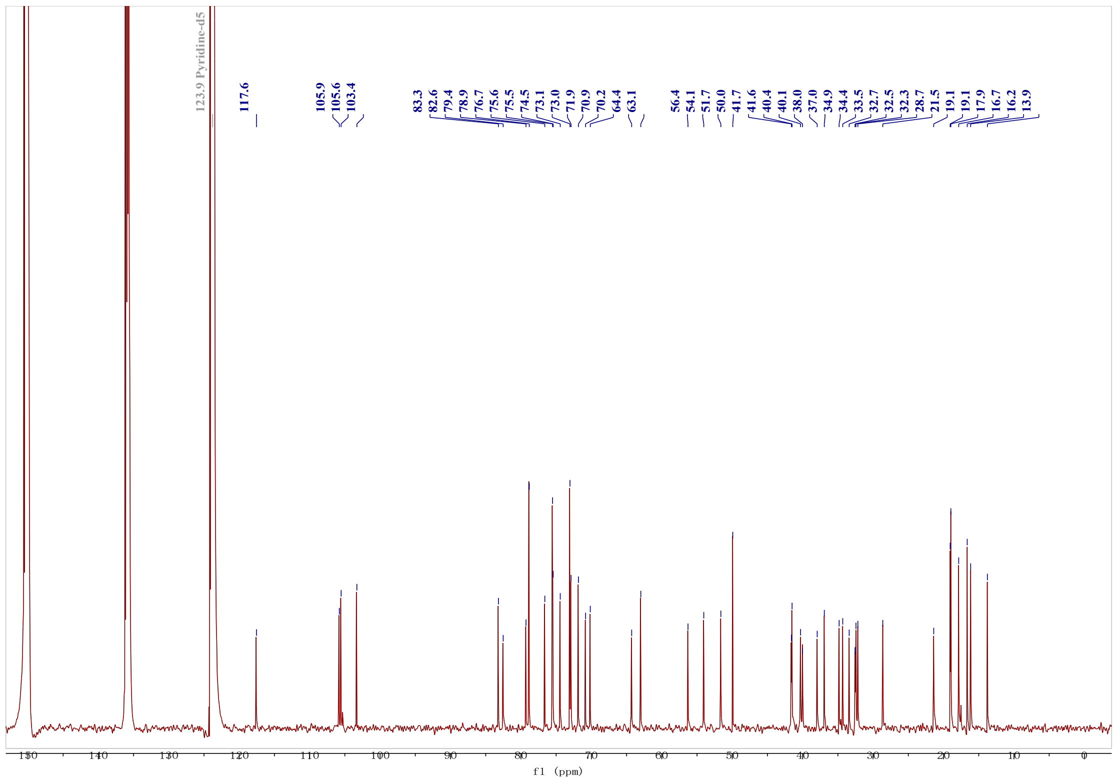

Figure S7. HSQC spectrum of compound **1**

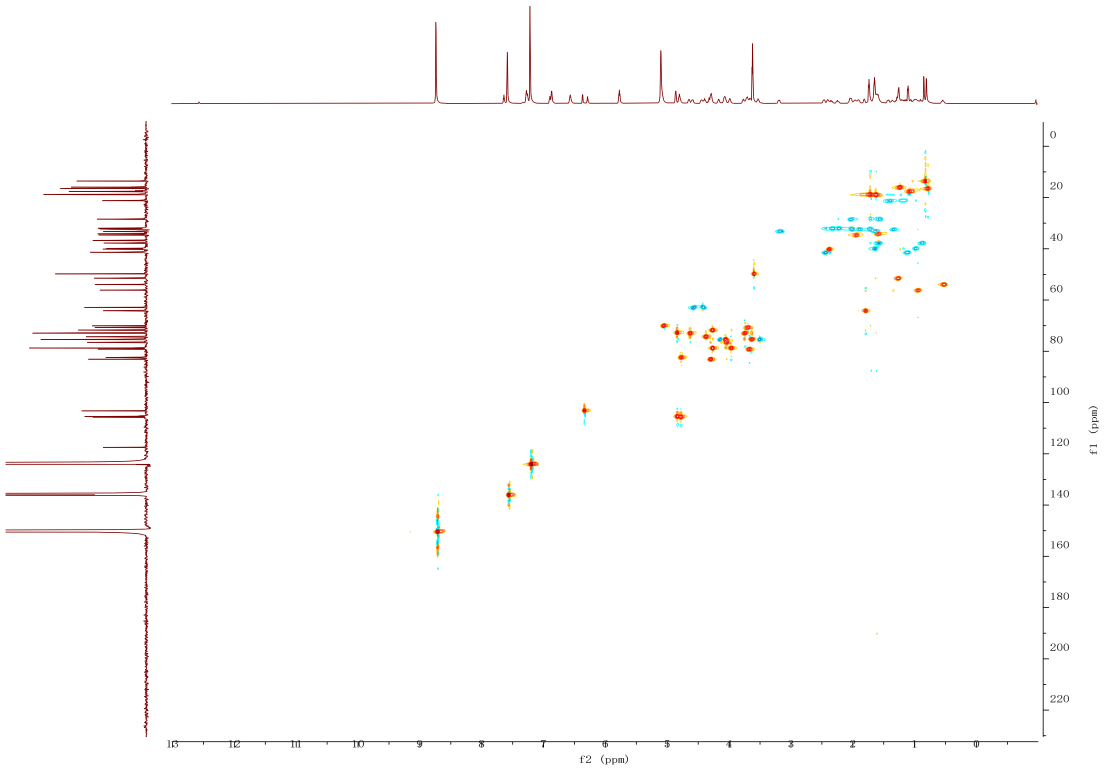

Figure S8. HMBC spectrum of compound **1**

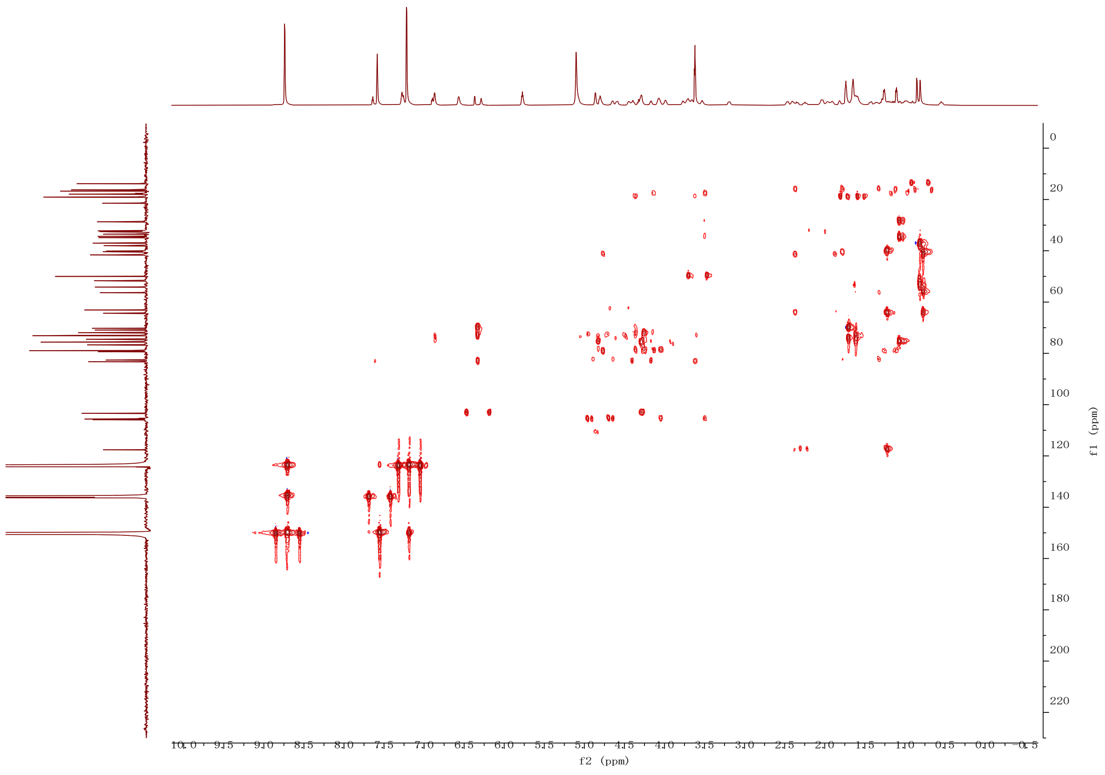

Figure S9. ^{1}H-^{1}H COSY spectrum of compound **1**

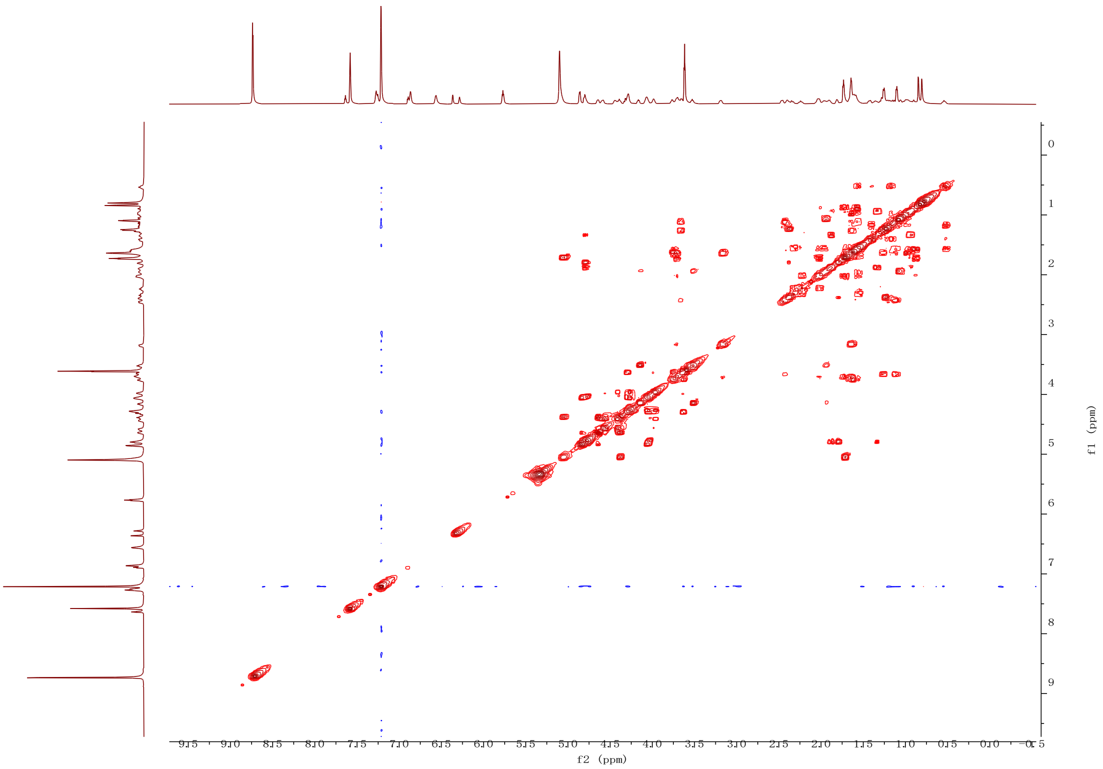

Figure S10. ROESY spectrum of compound **1**

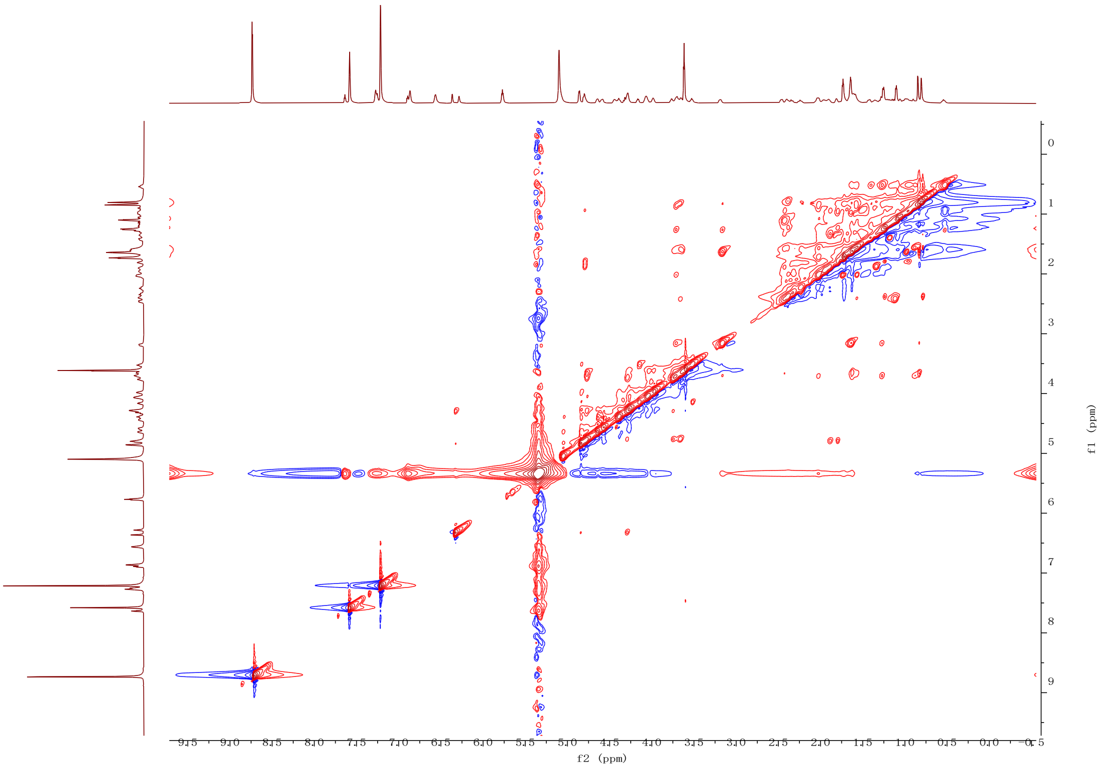

Figure S11. HR-ESI-MS Spectrum of compound **2**

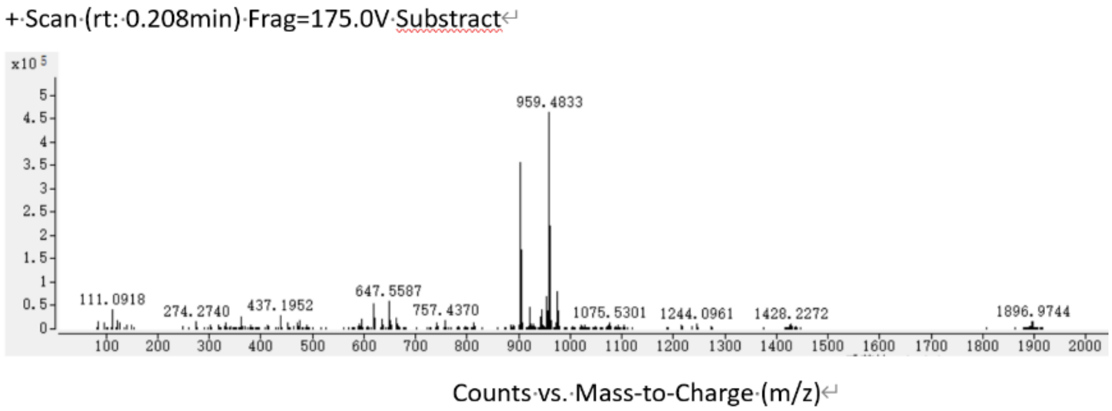

Figure S12. UV spectrum of compound **2**

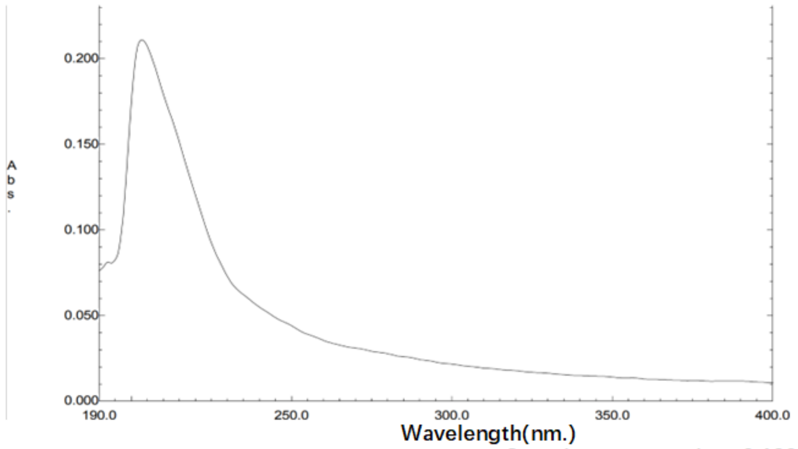

Sample concentration 0.1909 mg/ml
solvent: methanol

Created in: 18:41 2023/6/8
data: primeval

Measurement mode: Abs.
Scanning speed: Medium speed
Slit: 5
Sampling interval: 0.5

	Wavelength(nm.)	Abs.
1	203.5	0.2108

Figure S13. IR spectrum of compound **2**

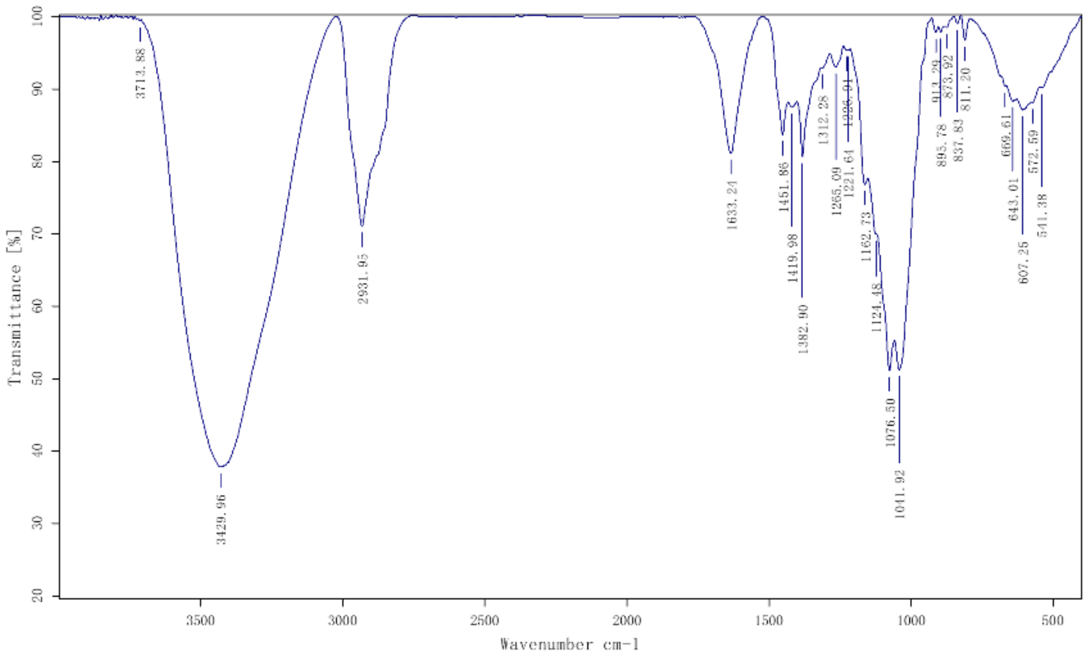

Figure S14. Optical rotations spectrum of compound **2**

Rudolph Research Analytical

This sample was measured on an Autopol VI, Serial #91058
Manufactured by Rudolph Research Analytical, Hackettstown, NJ, USA.

Measurement Date : Thursday, 14-OCT-2021

Set Temperature : OFF

Time Delay : Disabled

Delay between Measurement : Disabled

n	Average	Std.Dev.	% RSD	Maximum	Minimum
5	-43.11	1.45	-3.36	-42.22	-45.56

S.No	Sample ID	Time	Result	Scale	OR °Arc	WLG.nm	Lg.mm	Conc.g/100ml	Temp.
1	zmy-4	05:11:16 PM	-43.33	SR	-0.039	589	100.00	0.090	22.9
2	zmy-4	05:11:23 PM	-42.22	SR	-0.038	589	100.00	0.090	22.9
3	zmy-4	05:11:30 PM	-45.56	SR	-0.041	589	100.00	0.090	22.9
4	zmy-4	05:11:37 PM	-42.22	SR	-0.038	589	100.00	0.090	23.0
5	zmy-4	05:11:43 PM	-42.22	SR	-0.038	589	100.00	0.090	23.0

Figure S15. 1H-NMR spectrum of compound 2

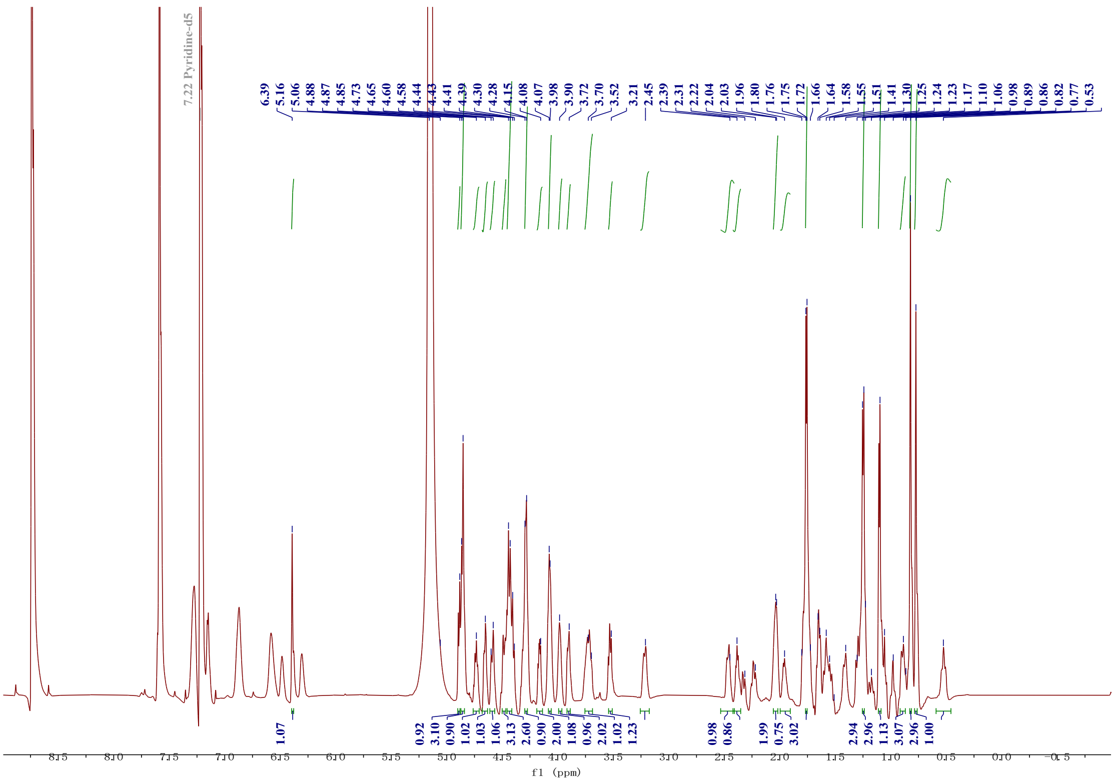

Figure S16. ^{13}C-NMR spectrum of compound **2**

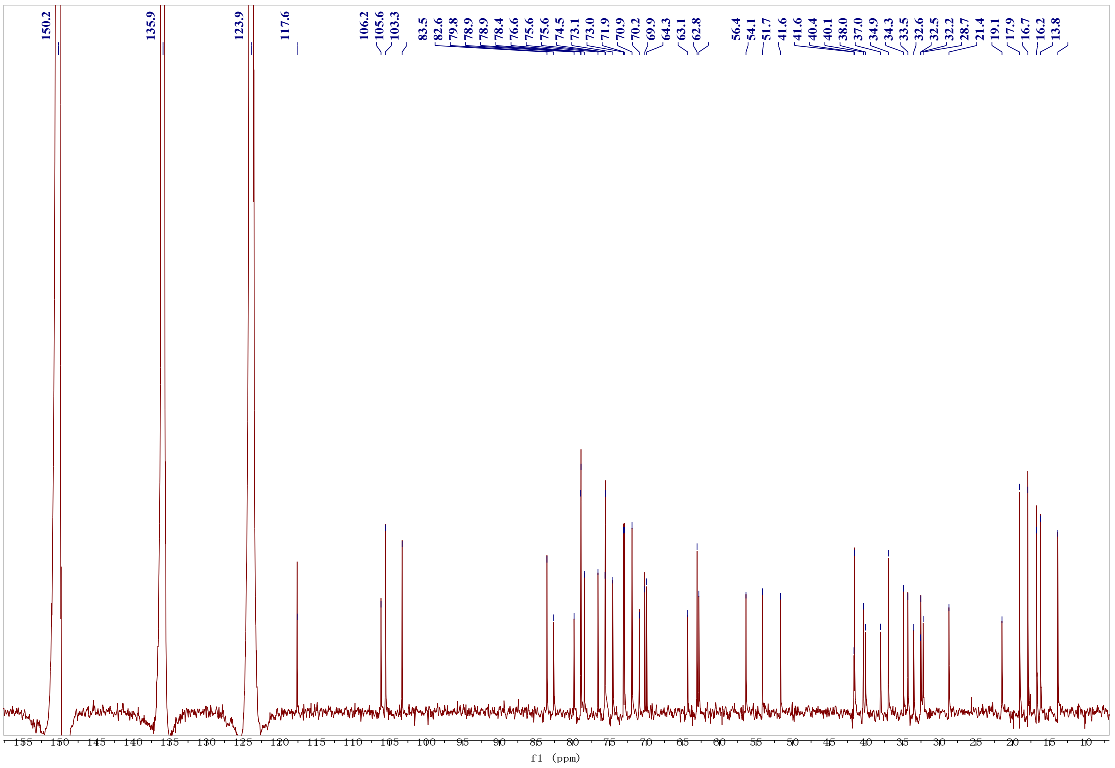

Figure S17. HSQC spectrum of compound **2**

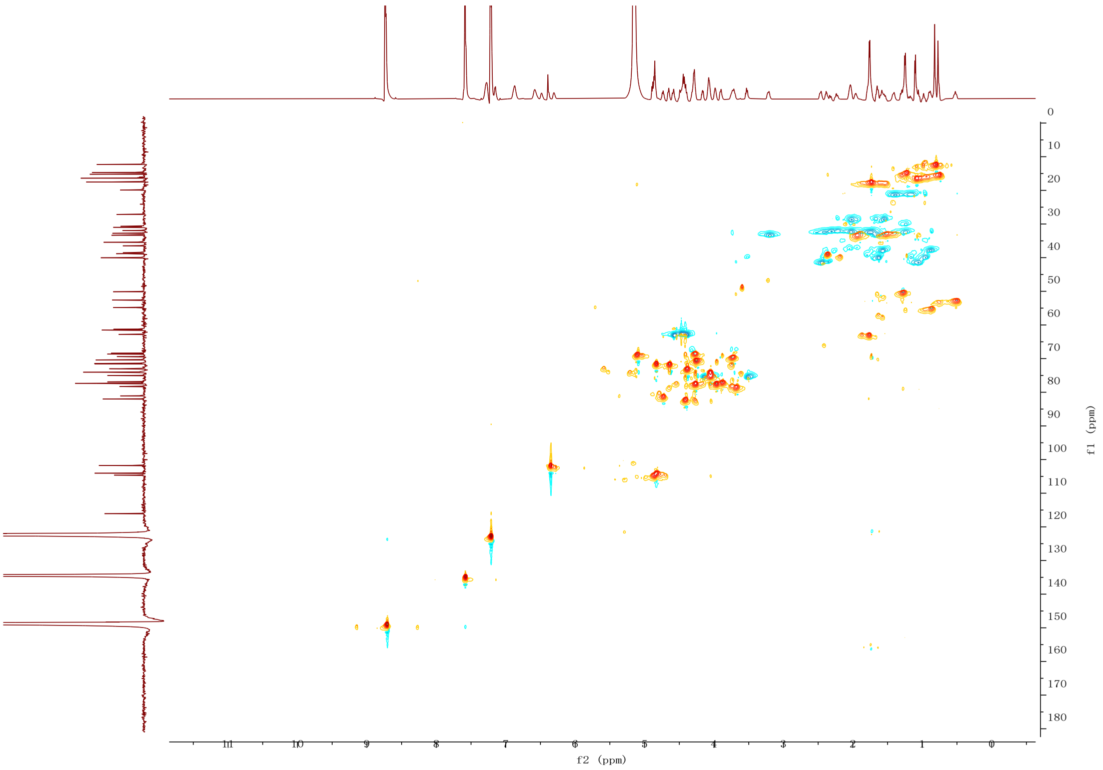

Figure S18. HMBC spectrum of compound **2**

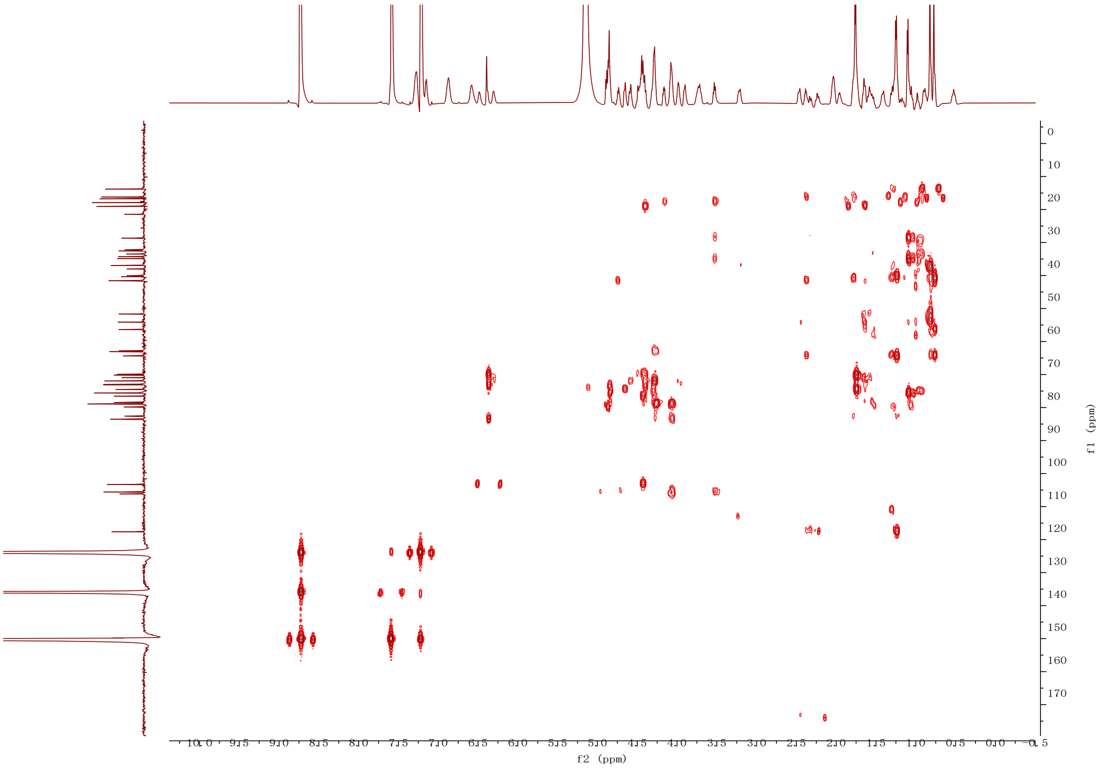

Figure S19. HR-ESI-MS Spectrum of compound **3**

+ Scan (rt: 0.206min) Frag=175.0V Substract

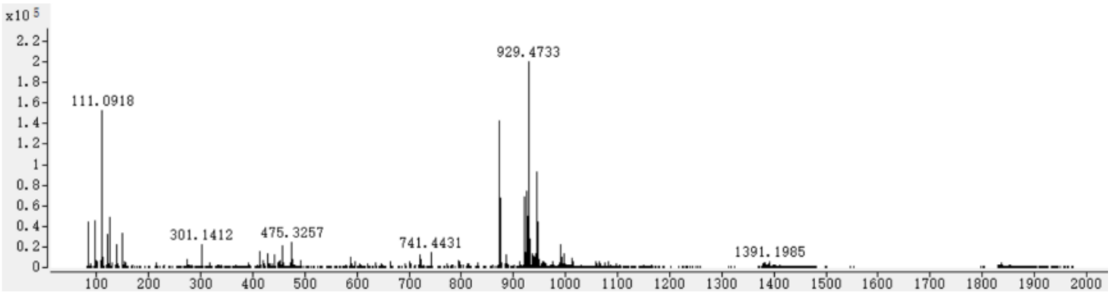

Counts vs. Mass-to-Charge (m/z)

Figure S20. UV spectrum of compound **3**

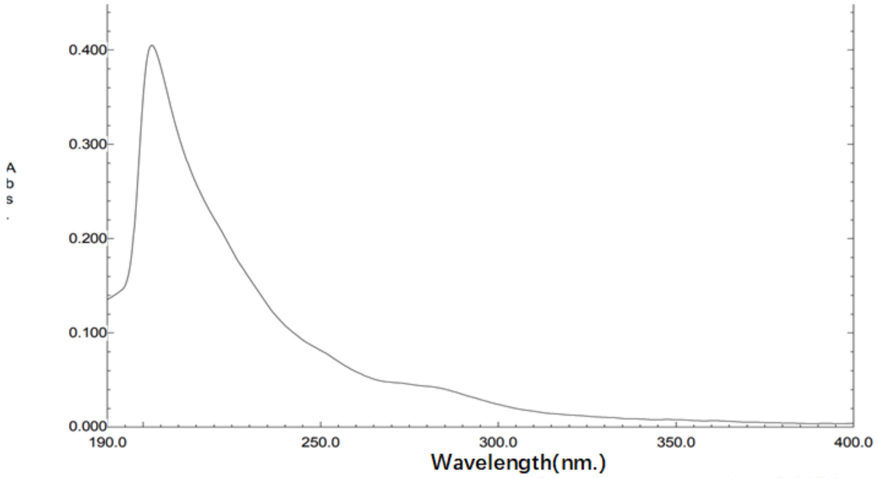

			Sample concentration	0.1154 mg/ml
Created in:	13:40	2023/6/6	solvent:	methanol
data:	primeval			

Measurement mode: Abs.
Scanning speed: Medium speed
Slit: 5
Sampling interval: 0.2

	Wavelength(nm.)	Abs.
1	202.2	0.4041
2	246	0.0898
3	269.6	0.0476

Figure S21. IR spectrum of compound **3**

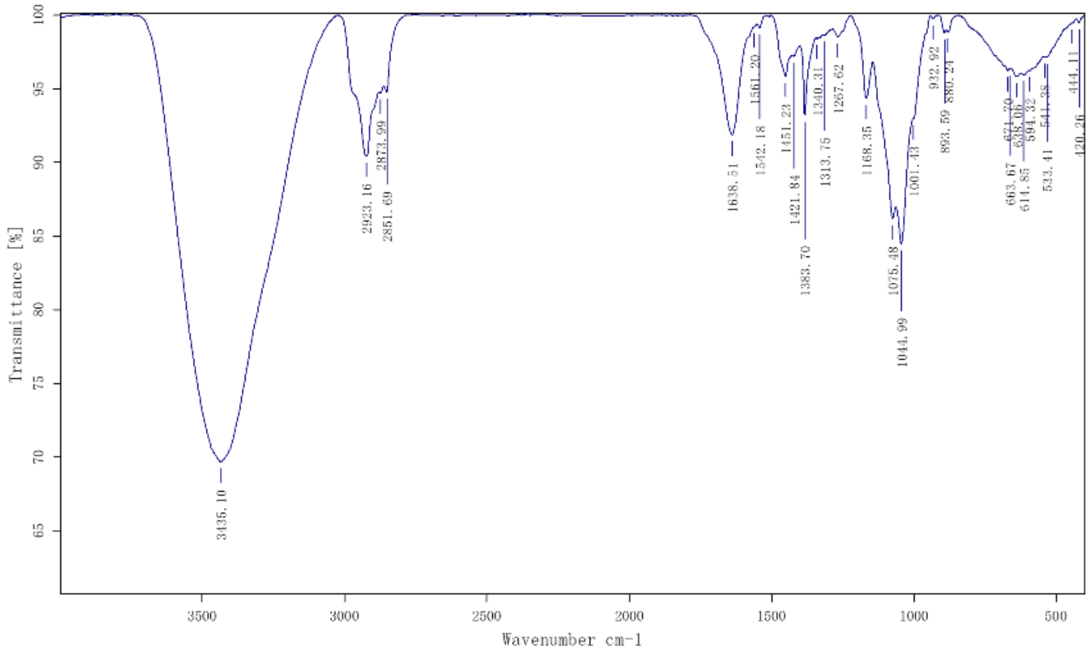

Figure S22. Optical rotations spectrum of compound **3**

Rudolph Research Analytical

This sample was measured on an Autopol VI, Serial #91058
Manufactured by Rudolph Research Analytical, Hackettstown, NJ, USA.

Measurement Date : Thursday, 14-OCT-2021

Set Temperature : OFF

Time Delay : Disabled

Delay between Measurement : Disabled

n	Average	Std.Dev.	% RSD	Maximum	Minimum
5	-20.00	0.00	0.00	-20.00	-20.00

S.No	Sample ID	Time	Result	Scale	OR °Arc	WLG.nm	Lg.mm	Conc.g/100ml	Temp.
1	zmy-6	04:36:16 PM	-20.00	SR	-0.016	589	100.00	0.080	23.1
2	zmy-6	04:36:22 PM	-20.00	SR	-0.016	589	100.00	0.080	23.1
3	zmy-6	04:36:29 PM	-20.00	SR	-0.016	589	100.00	0.080	23.1
4	zmy-6	04:36:36 PM	-20.00	SR	-0.016	589	100.00	0.080	23.1
5	zmy-6	04:36:43 PM	-20.00	SR	-0.016	589	100.00	0.080	23.1

Figure S23. ^{1}H-NMR spectrum of compound **3**

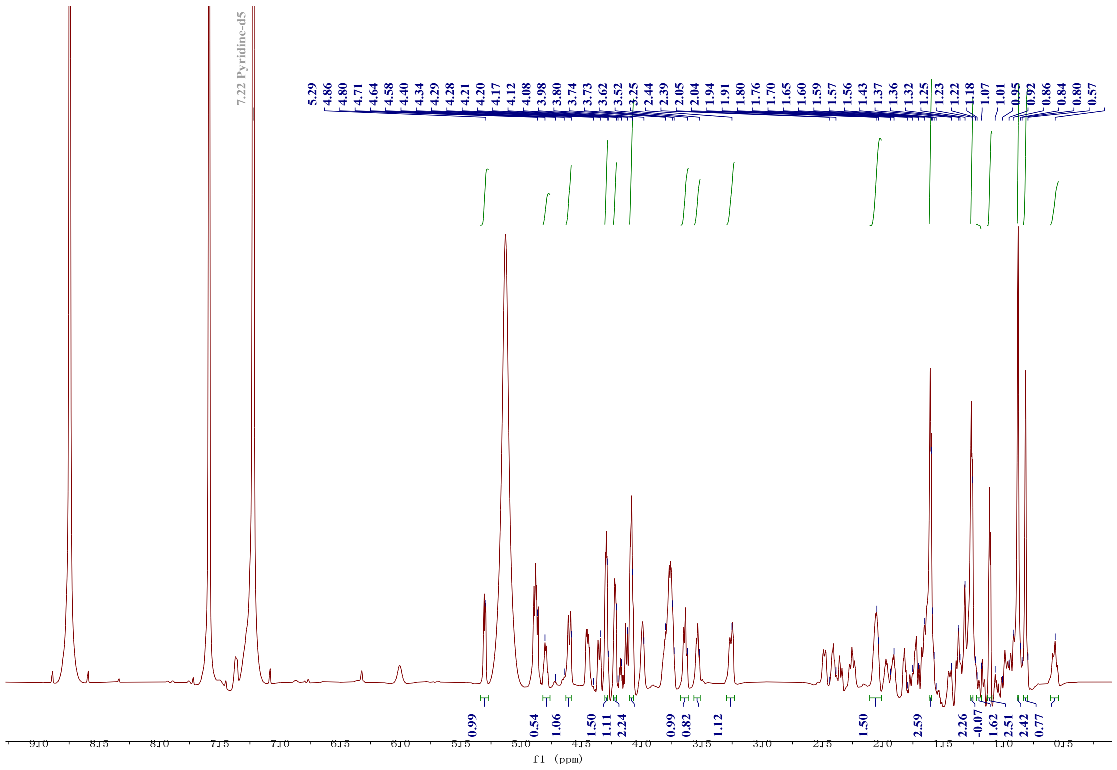

Figure S24. ^{13}C-NMR spectrum of compound **3**

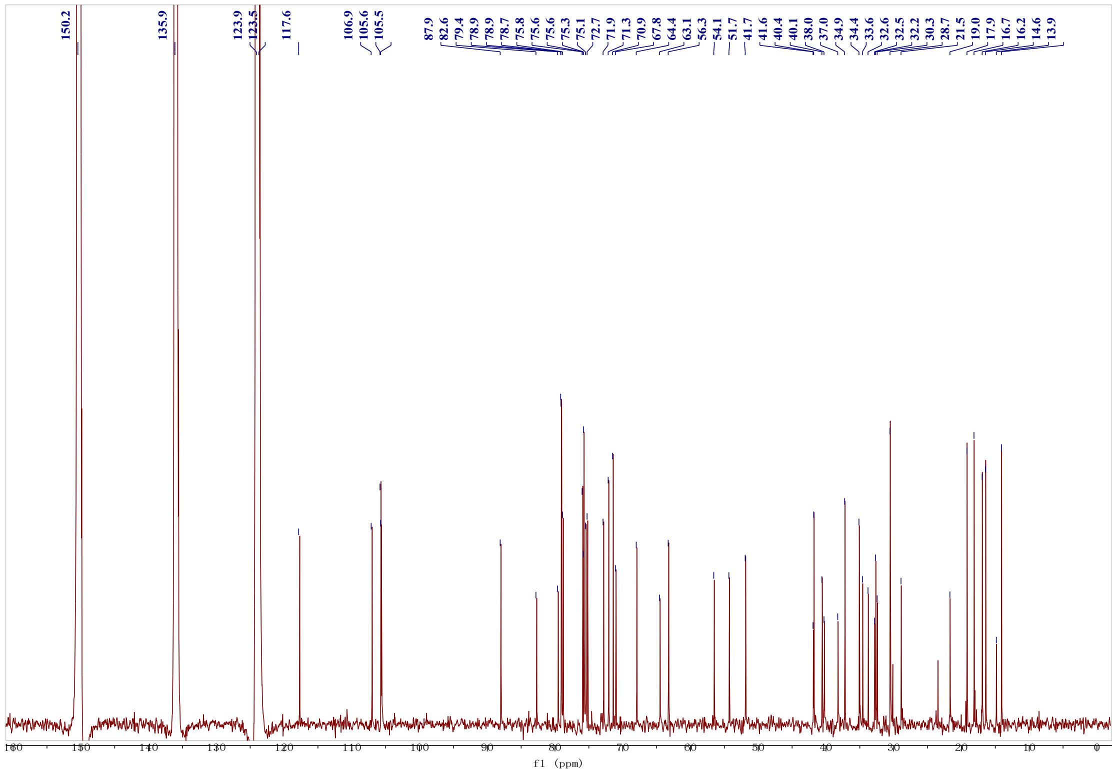

Figure S25. HSQC spectrum of compound **3**

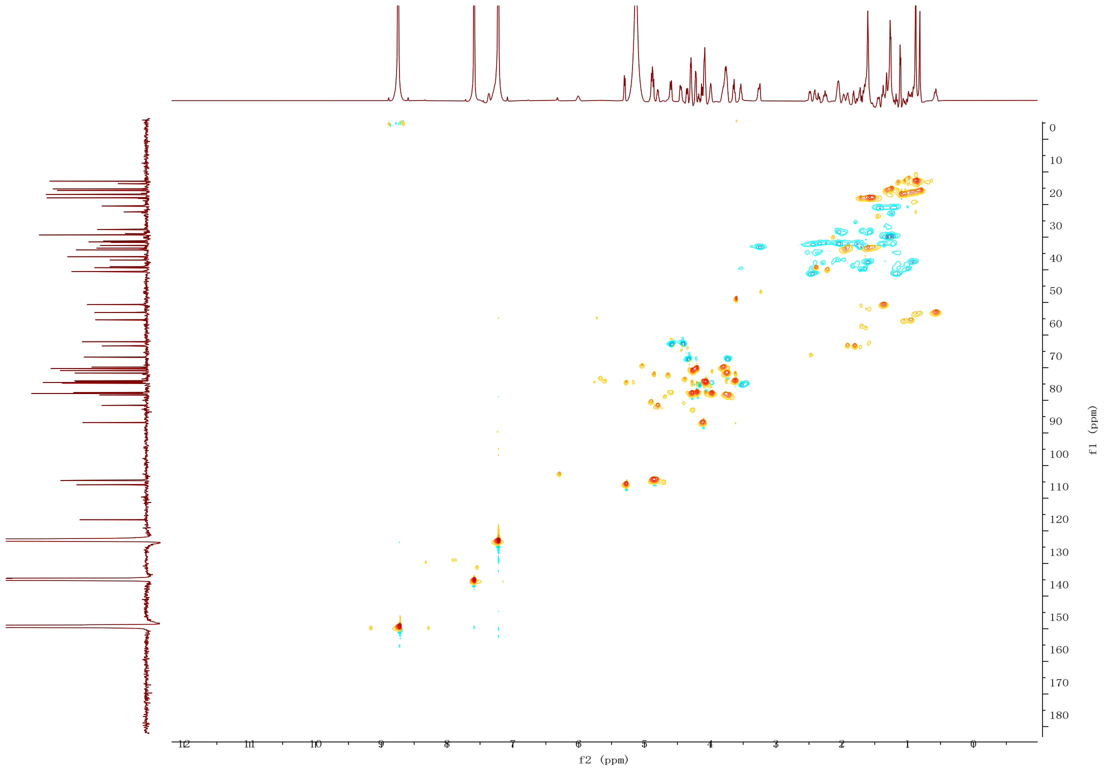

Figure S26. HMBC spectrum of compound **3**

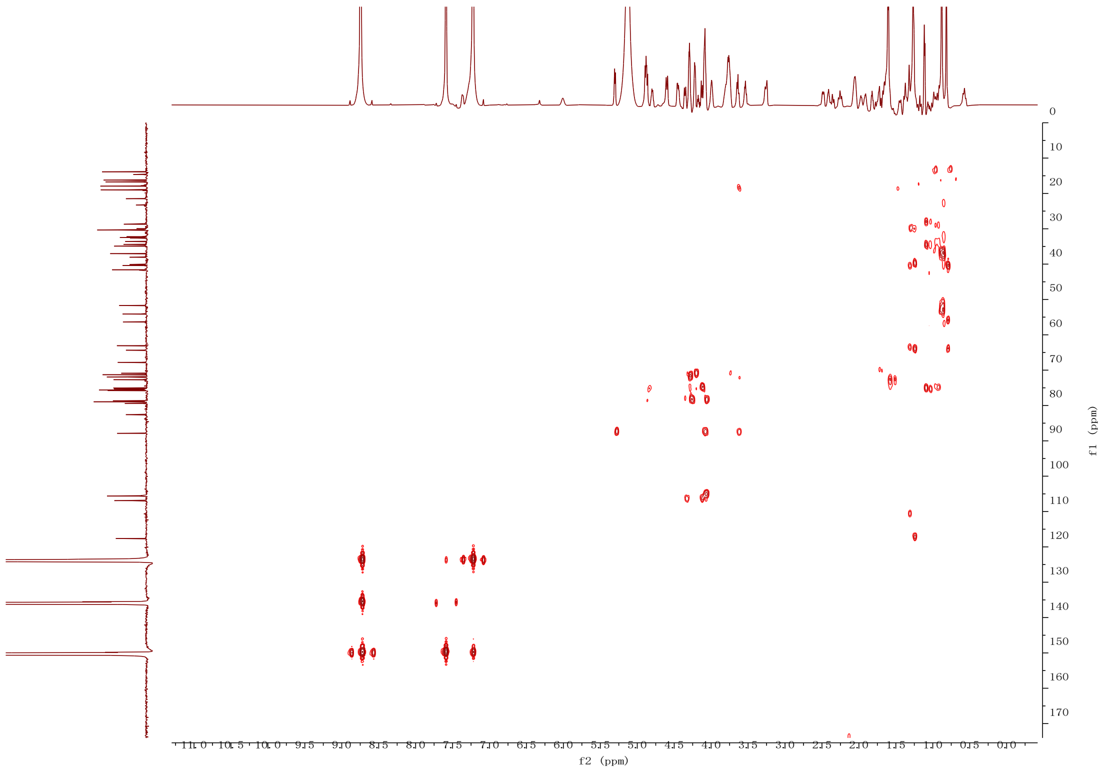

Figure S27. HR-ESI-MS Spectrum of compound **4**

+ Scan (rt: 0.226min) Frag=175.0V Substract

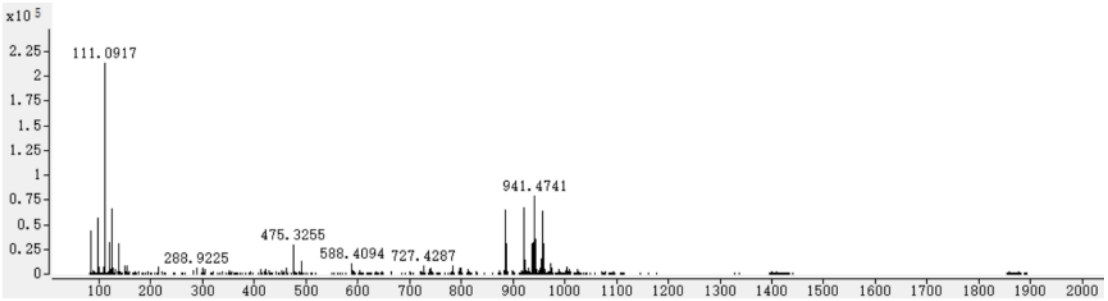

Counts vs. Mass-to-Charge (m/z)

Figure S28. UV spectrum of compound **4**

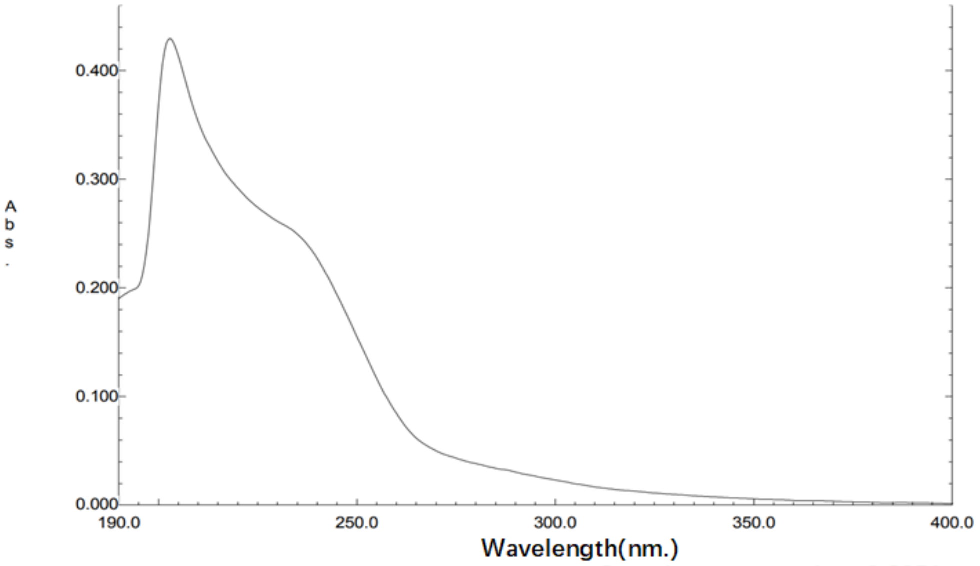

Created in: 18:52 2023/6/8

data: primeval

Sample concentration 0.2851 mg/ml

solvent: methanol

Measurement mode: Abs.

Scanning speed: Medium speed

Slit: 5

Sampling interval: 0.5

	Wavelength(nm.)	Abs.
1	203	0.4293
2	224.5	0.2755
3	273	0.0454

Figure S29. IR spectrum of compound **4**

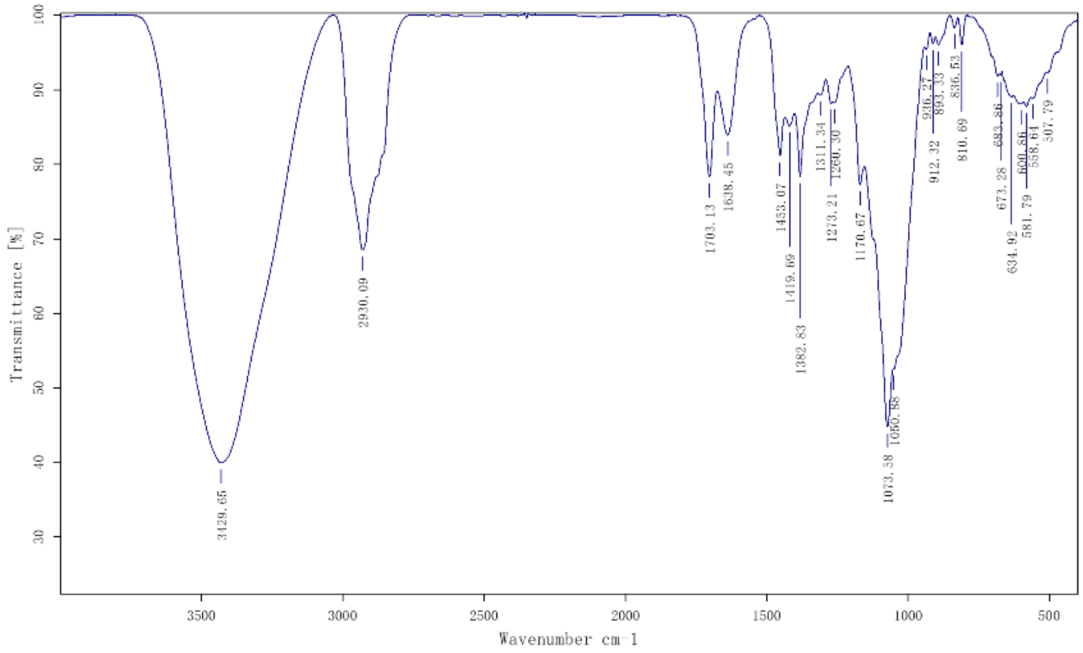

Figure S30. Optical rotations spectrum of compound **4**

Rudolph Research Analytical

This sample was measured on an Autopol VI, Serial #91058
Manufactured by Rudolph Research Analytical, Hackettstown, NJ, USA.

Measurement Date : Thursday, 14-OCT-2021

Set Temperature : OFF

Time Delay : Disabled

Delay between Measurement : Disabled

n	Average	Std.Dev.	% RSD	Maximum	Minimum
5	-41.78	0.61	-1.46	-41.11	-42.22

S.No	Sample ID	Time	Result	Scale	OR °Arc	WLG.nm	Lg.mm	Conc.g/100ml	Temp.
1	zmy-5	05:00:35 PM	-42.22	SR	-0.038	589	100.00	0.090	23.0
2	zmy-5	05:00:42 PM	-41.11	SR	-0.037	589	100.00	0.090	23.0
3	zmy-5	05:00:49 PM	-42.22	SR	-0.038	589	100.00	0.090	23.0
4	zmy-5	05:00:55 PM	-42.22	SR	-0.038	589	100.00	0.090	23.0
5	zmy-5	05:01:02 PM	-41.11	SR	-0.037	589	100.00	0.090	23.0

Figure S31. ^{1}H-NMR spectrum of compound **4**

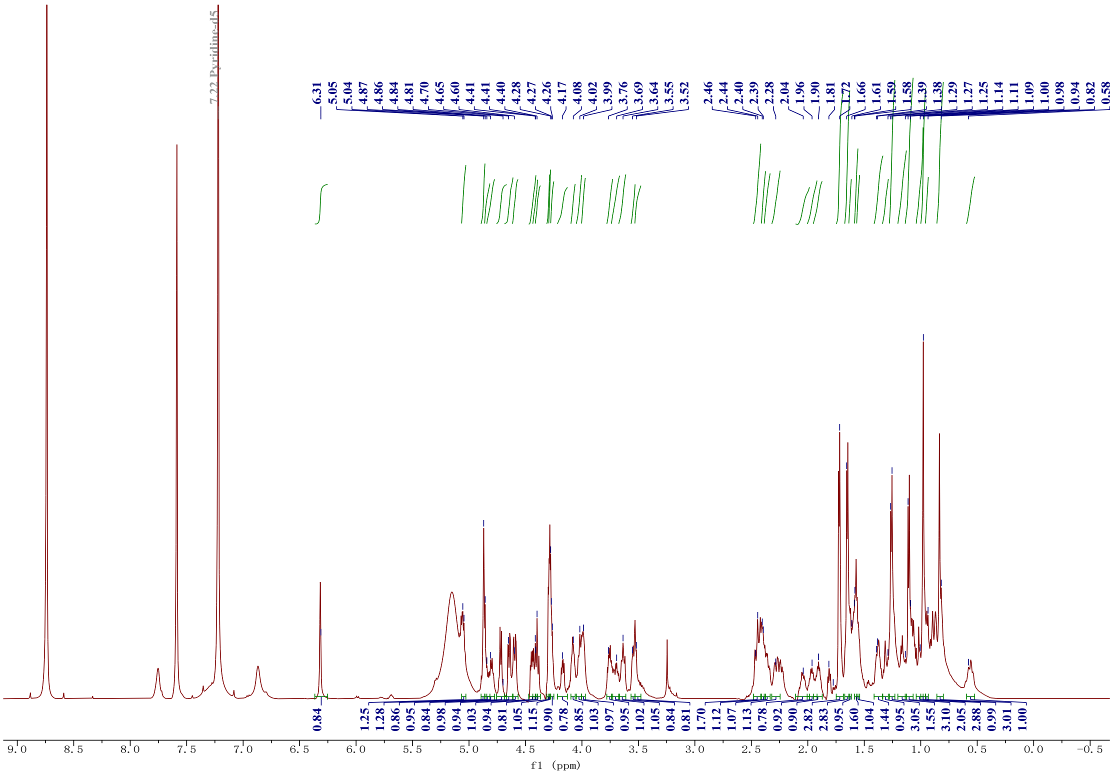

Figure S32. ^{13}C-NMR spectrum of compound **4**

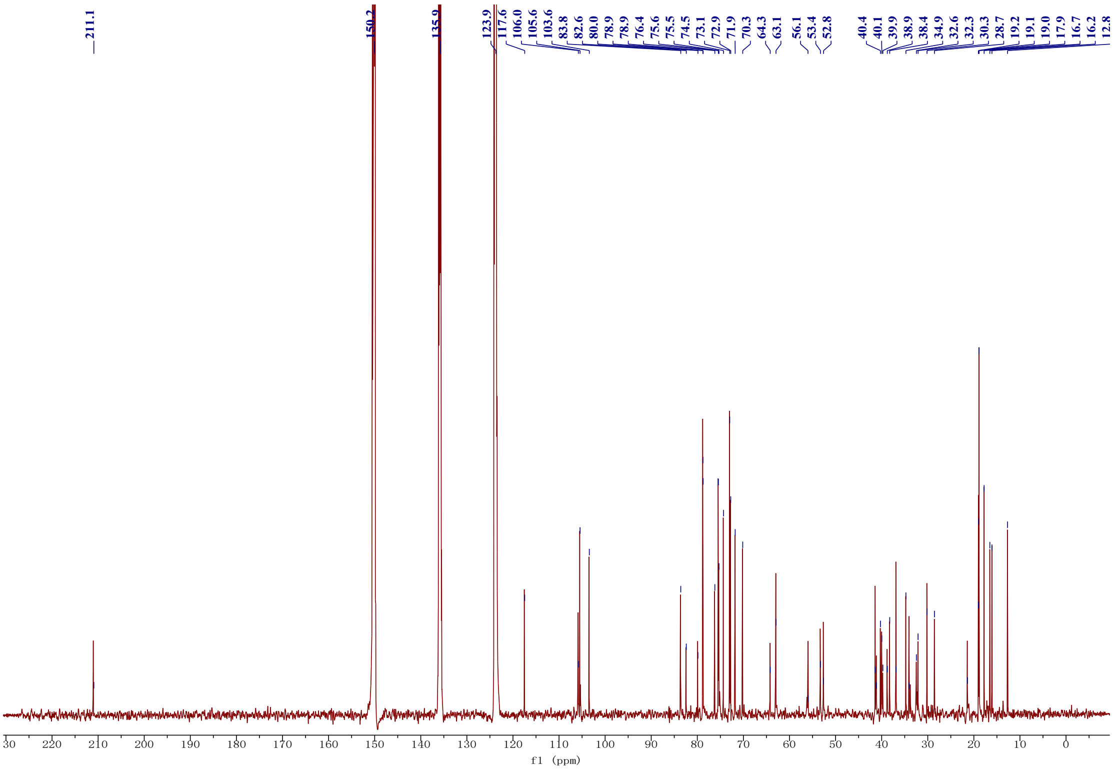

Figure S33. HSQC spectrum of compound **4**

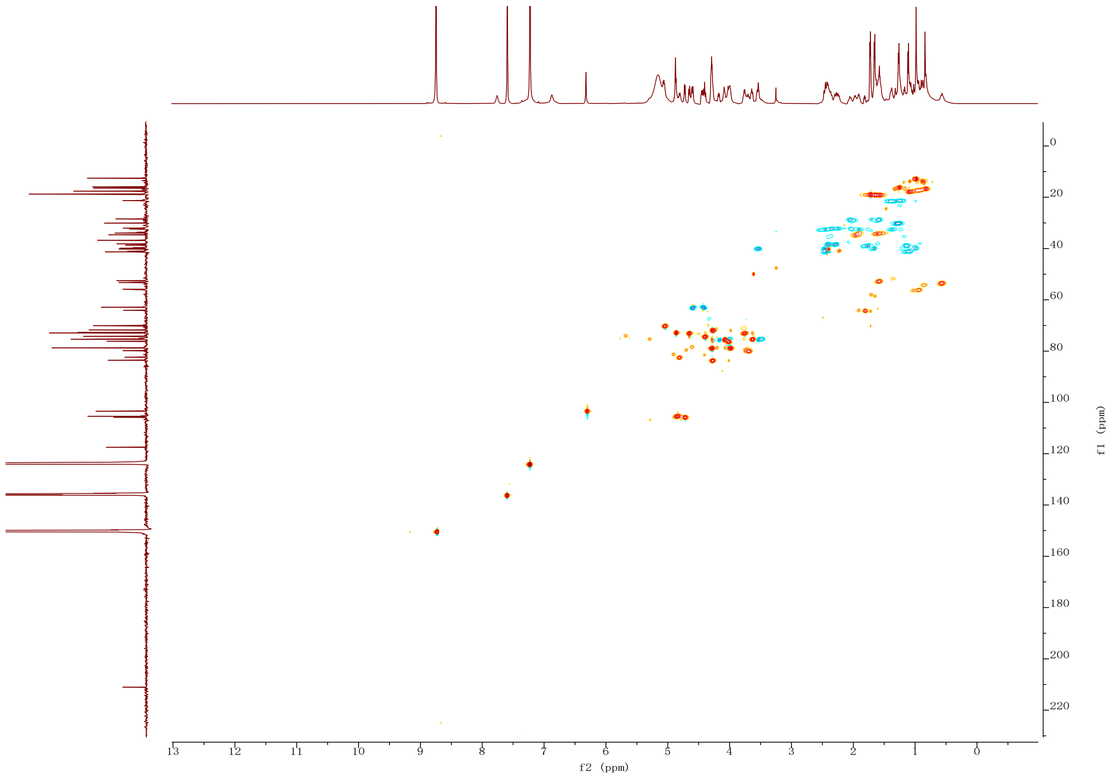

Figure S34. HMBC spectrum of compound **4**

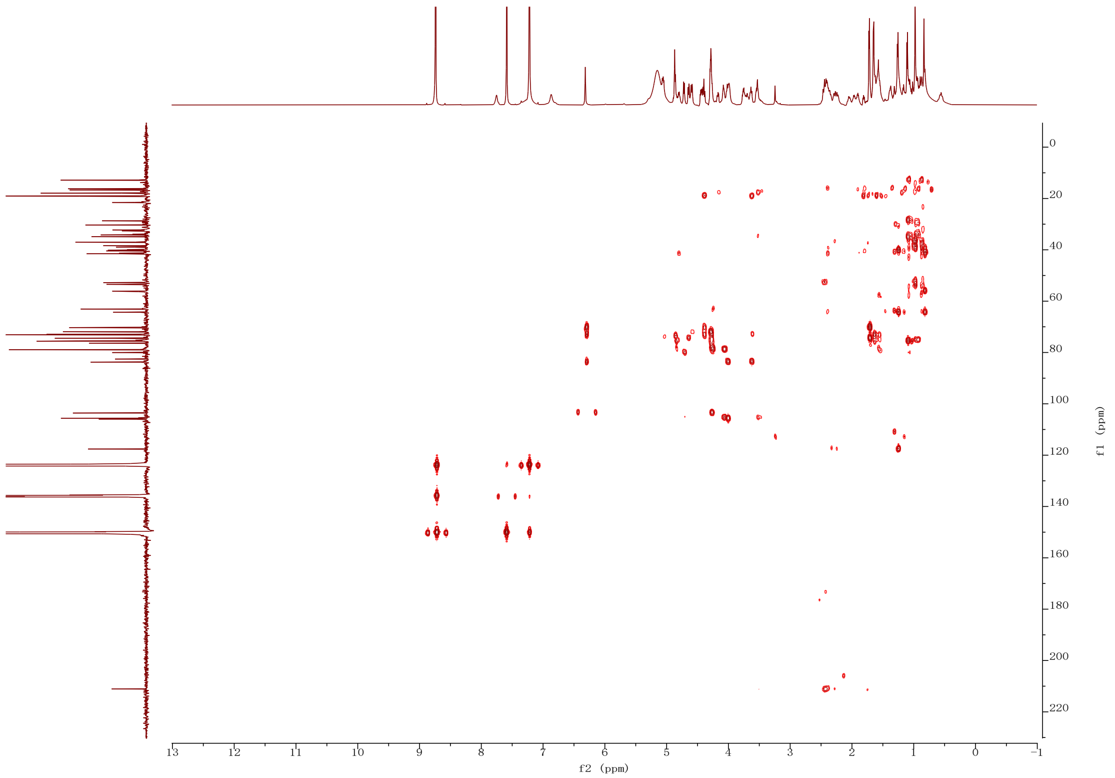

Figure S35. HR-ESI-MS Spectrum of compound **5**

+ Scan (rt: 0.143min) Frag=175.0V Substract

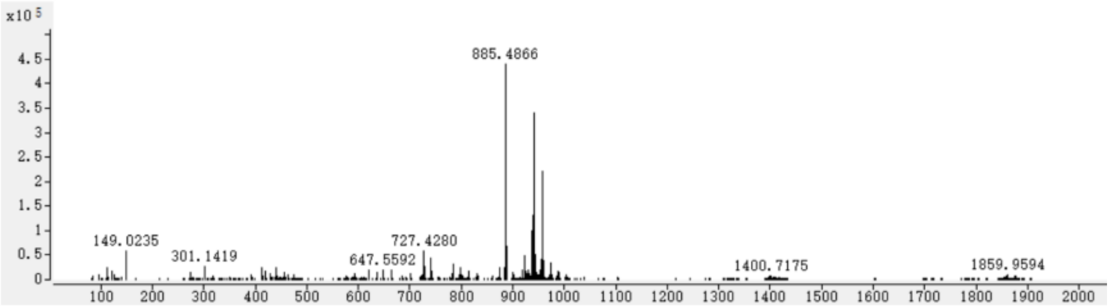

Counts vs. Mass-to-Charge (m/z)

Figure S36. UV Spectrum of compound **5**

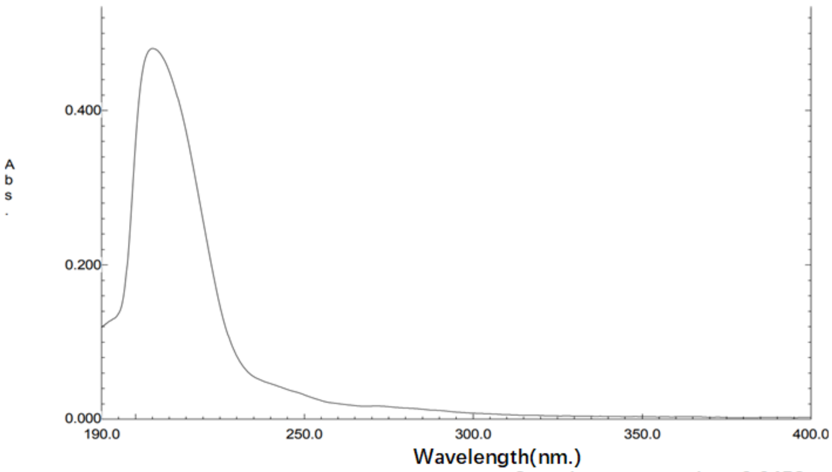

			Sample concentration	0.0458 mg/ml
Created in:	13:15	2023/6/6	solvent:	methanol
data:	primeval			

Measurement mode:	Abs.
Scanning speed:	Medium speed
Slit:	5
Sampling interval:	0.2

	Wavelength(nm.)	Abs.
1	205.6	0.4797
2	239.2	0.0472
3	270.4	0.017

Figure S37. IR spectrum of compound **5**

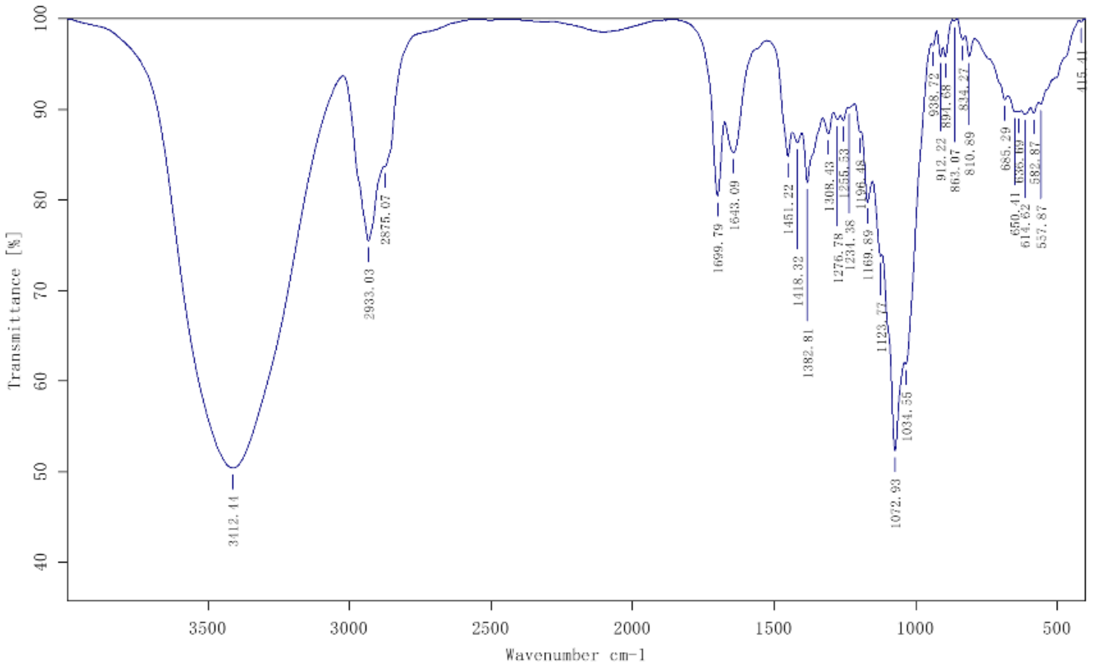

Figure S38. Optical rotations spectrum of compound **5**

Rudolph Research Analytical

This sample was measured on an Autopol VI, Serial #91058
Manufactured by Rudolph Research Analytical, Hackettstown, NJ, USA.

Measurement Date : Tuesday, 06-JUN-2023

Set Temperature : OFF

Time Delay : Disabled

Delay between Measurement : Disabled

n	Average	Std.Dev.	% RSD	Maximum	Minimum
5	-24.30	0.39	-1.60	-23.70	-24.80

S.No	Sample ID	Time	Result	Scale	OR °Arc	WLG.nm	Lg.mm	Conc.g/100ml	Temp.
1	XST-01	11:33:31 AM	-24.80	SR	-0.0248	589	100.00	0.100	25.7
2	XST-01	11:33:39 AM	-24.30	SR	-0.0243	589	100.00	0.100	25.7
3	XST-01	11:33:48 AM	-24.40	SR	-0.0244	589	100.00	0.100	25.7
4	XST-01	11:33:56 AM	-23.70	SR	-0.0237	589	100.00	0.100	25.7
5	XST-01	11:34:04 AM	-24.30	SR	-0.0243	589	100.00	0.100	25.7

Figure S39. ^{1}H-NMR spectrum of compound **5**

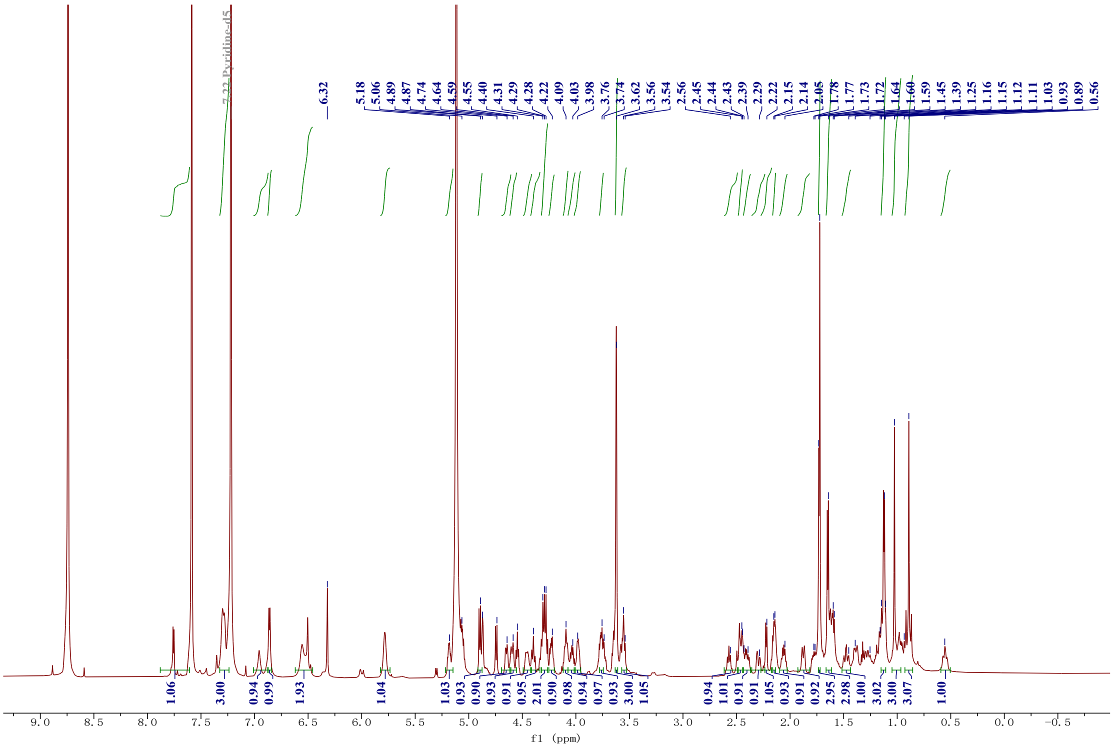

Figure S40. ^{13}C-NMR spectrum of compound **5**

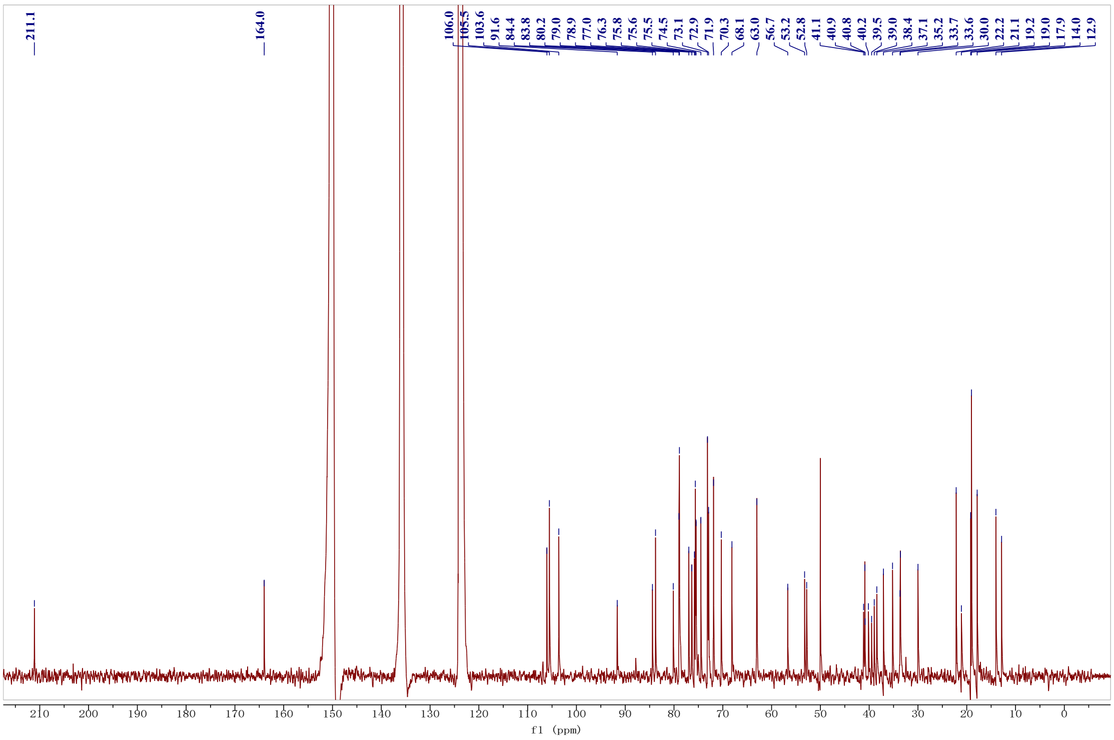

Figure S41. HSQC spectrum of compound **5**

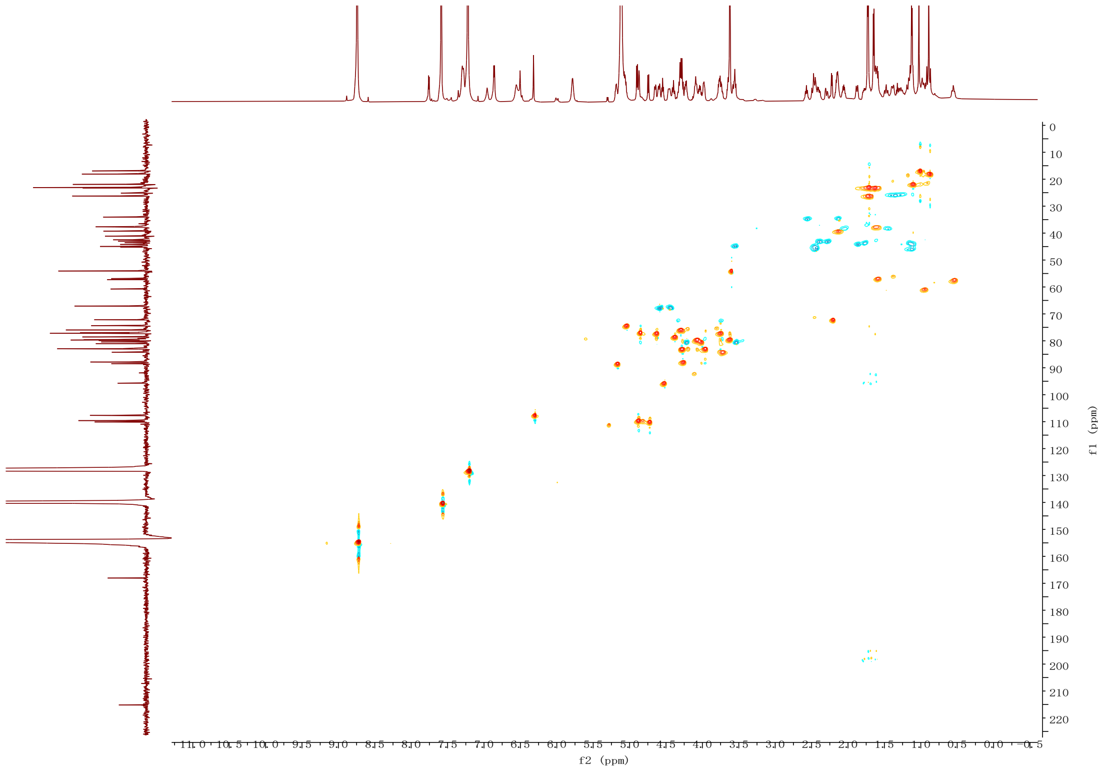

Figure S42. HMBC spectrum of compound **5**

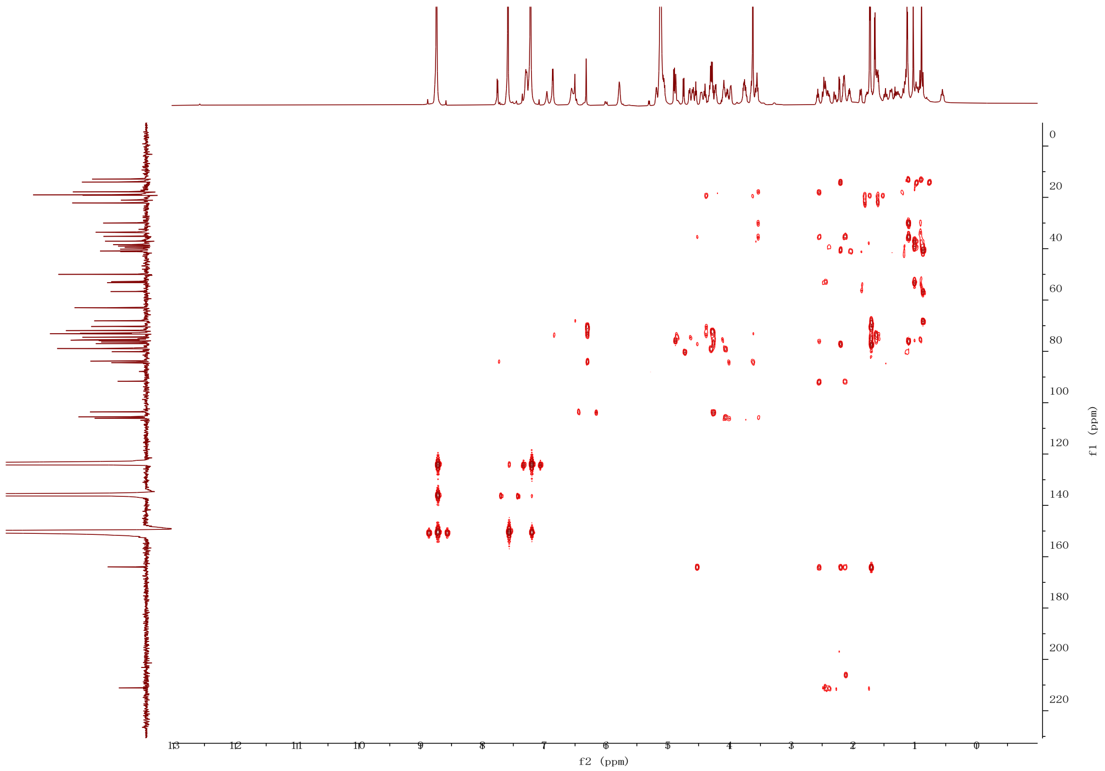

Figure S43. HR-ESI-MS Spectrum of compound **6**

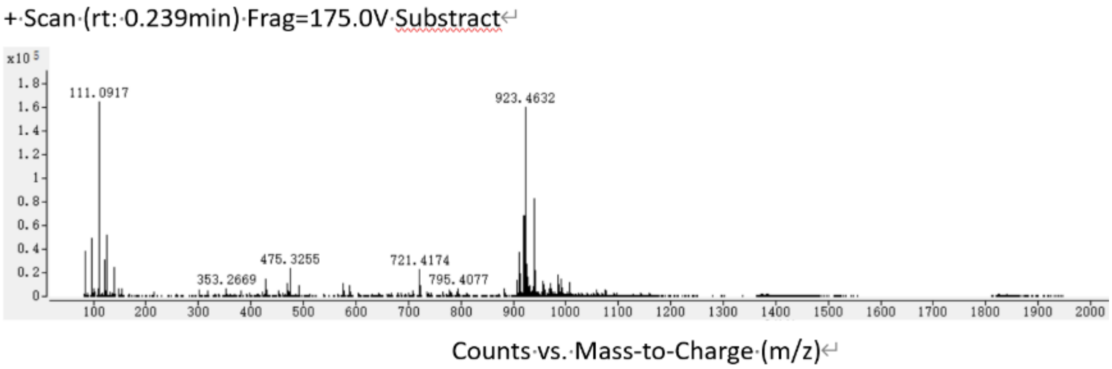

Figure S44. UV spectrum of compound **6**

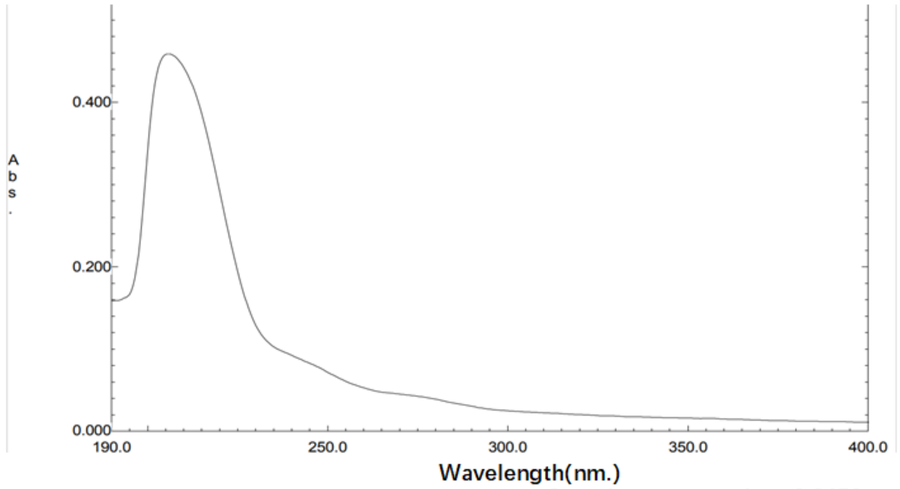

			Sample concentration	0.0452 mg/ml
Created in:	18:15	2023/6/8	solvent:	methanol
data:	primeval			

Measurement mode: Abs.
Scanning speed: Medium speed
Slit: 5
Sampling interval: 0.5

	Wavelength(nm.)	Abs.
1	206	0.4587
2	233.5	0.1078
3	265	0.0473

Figure S45. IR spectrum of compound **6**

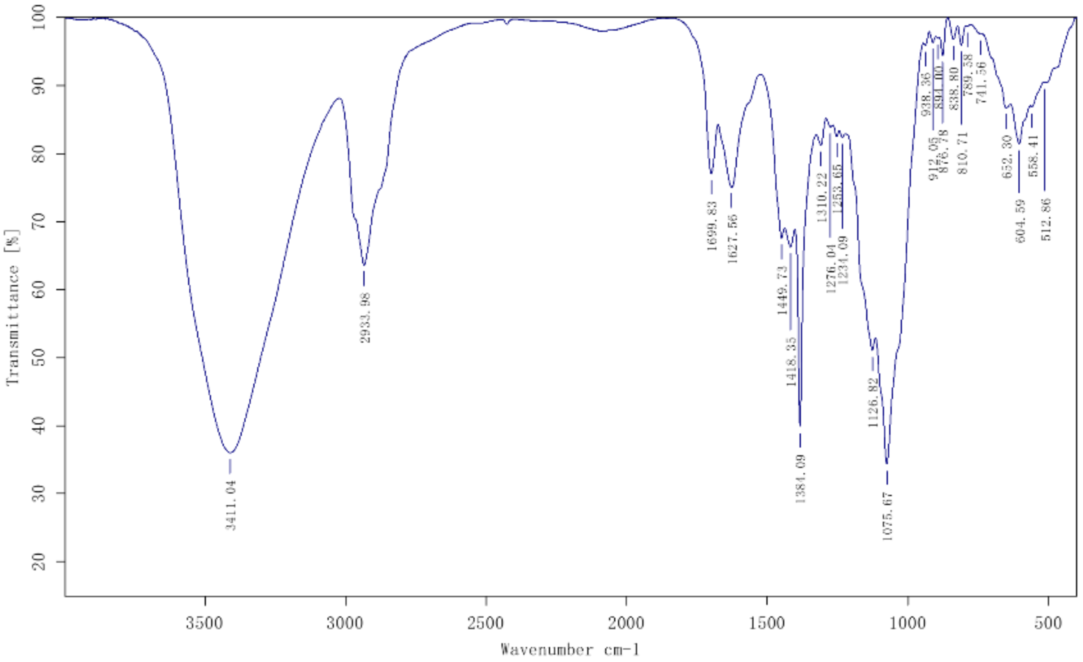

Figure S46. Optical rotations spectrum of compound **6**

Rudolph Research Analytical

This sample was measured on an Autopol VI, Serial #91058
Manufactured by Rudolph Research Analytical, Hackettstown, NJ, USA.

Measurement Date : Thursday, 08-JUN-2023

Set Temperature : OFF

Time Delay : Disabled

Delay between Measurement : Disabled

n	Average	Std.Dev.	% RSD	Maximum	Minimum
5	-15.49	4.09	-26.40	-9.86	-19.43

S.No	Sample ID	Time	Result	Scale	OR °Arc	WLG.nm	Lg.mm	Conc.g/100ml	Temp.
1	XST-06	04:07:07 PM	-19.43	SR	-0.0136	589	100.00	0.070	25.8
2	XST-06	04:07:16 PM	-9.86	SR	-0.0069	589	100.00	0.070	25.8
3	XST-06	04:07:24 PM	-12.57	SR	-0.0088	589	100.00	0.070	25.8
4	XST-06	04:07:32 PM	-18.29	SR	-0.0128	589	100.00	0.070	25.8
5	XST-06	04:07:40 PM	-17.29	SR	-0.0121	589	100.00	0.070	25.8

Figure S47. ^{1}H-NMR spectrum of compound **6**

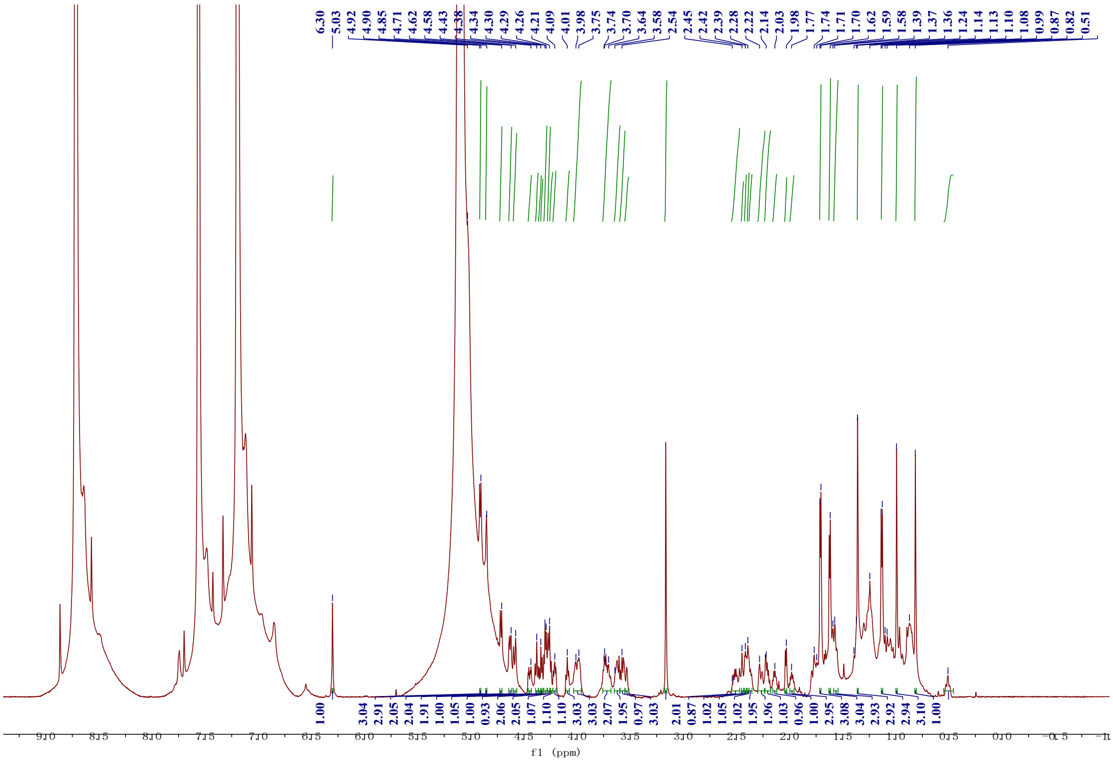

Figure S48. ^{13}C-NMR spectrum of compound **6**

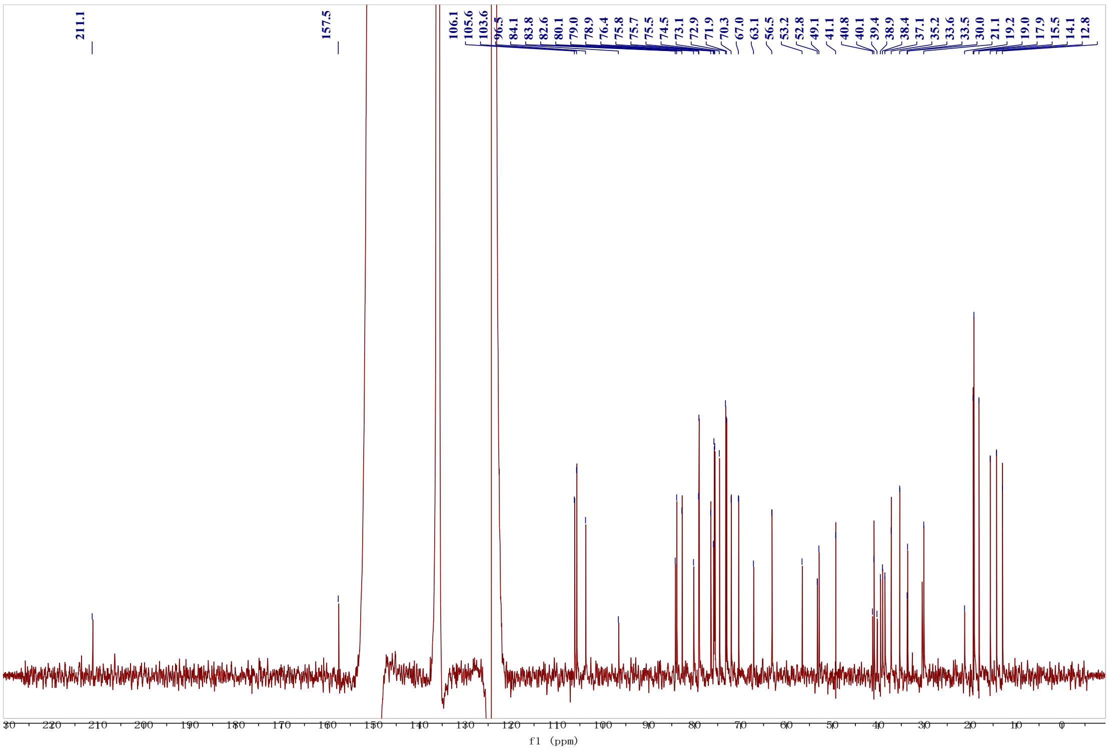

Figure S49. HSQC spectrum of compound **6**

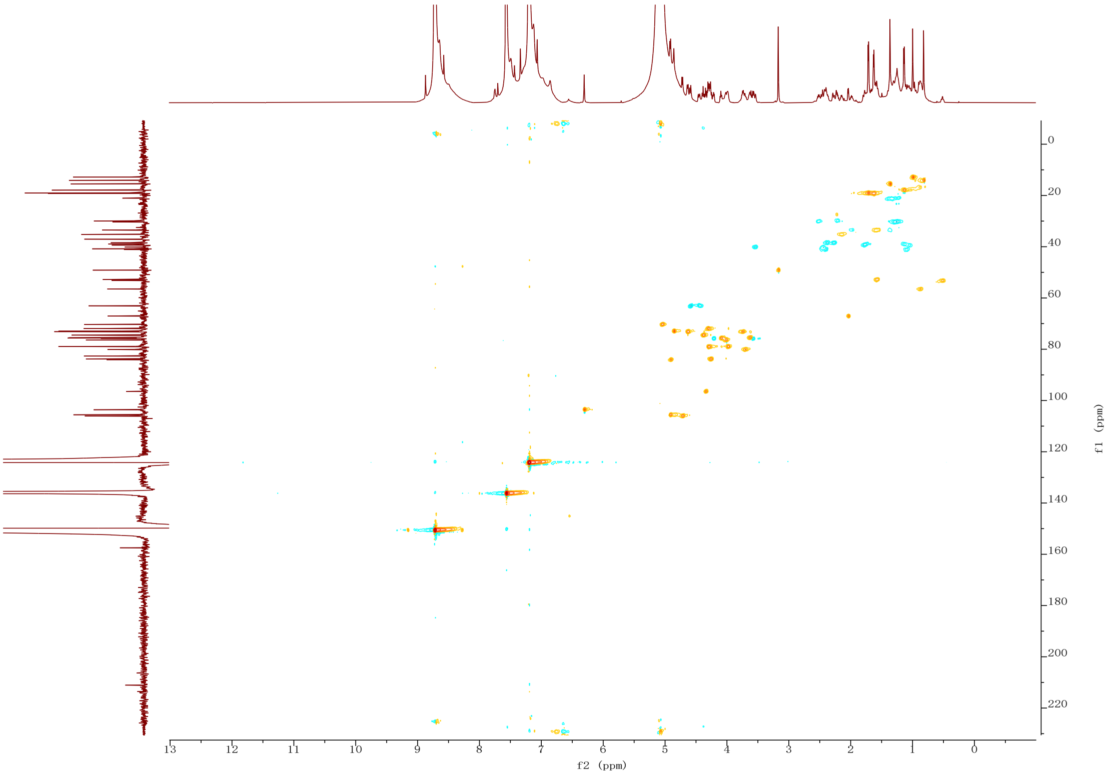

Figure S50. HMBC spectrum of compound **6**

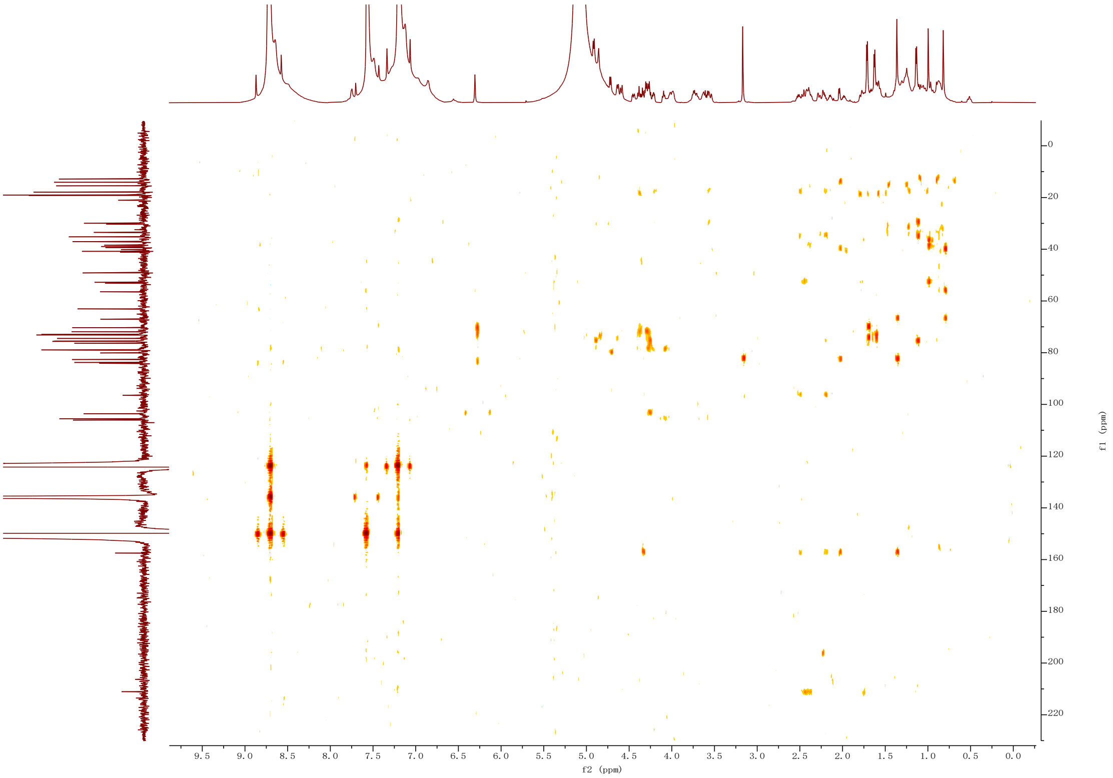

Supplement: Supplementary file 1 [file molecules-29-01316-s001.zip › molecules-2862031-supplementary.pdf]
